# A Metal-Free Regioselective Multicomponent Approach for the Synthesis of Free Radical Scavenging Pyrimido-Fused Indazoles and Their Fluorescence Studies

**DOI:** 10.3390/molecules21111571

**Published:** 2016-11-18

**Authors:** Jeyakannu Palaniraja, Selvaraj Mohana Roopan, G Mokesh Rayalu, Naif Abdullah Al-Dhabi, Mariadhas Valan Arasu

**Affiliations:** 1 Chemistry of Heterocycles & Natural Product Research Laboratory, Department of Chemistry, School of Advanced Sciences, VIT University, Vellore 632014, Tamilnadu, India; myne.palani@gmail.com; 2 G Mokesh Rayalu, Department of mathematics, School of Advanced Sciences, VIT University, Vellore 632014, Tamilnadu, India; mokesh.g@gmail.com; 3 Department of Botany and Microbiology, Addiriyah Chair for Environmental Studies, College of Science, King Saud University, P.O. Box 2455, Riyadh 11451, Saudi Arabia; naldhabi@ksu.edu.sa (N.A.A.-D.); mvalanarasu@gmail.com (M.V.A.)

**Keywords:** *N*-fused pyrimidine, Strecker synthesis, A^3^ coupling, Box-Behnken design (BBD), fluorescence

## Abstract

This study deals with a new and efficient metal-free regioselective synthesis of pyrimido-fused indazoles with nitrogen ring junction motifs. We have developed a metal-free domino type reaction between 3-aminoindazole, aryl aldehydes and aceotophenones in the presence of KOH/DMF that leads to pyrimido[1,2-*b*]indazole analogues. Response Surface Methodology (RSM) coupled with a Box-Behnken design (BBD) were utilized for exploring the effect of base used (A), temperature of reaction (B) and (C), reaction time. This approach can allow access to a variety of pyrimidoindazole fluorophores and related compounds. The compound *N,N*-dimethyl-4-(2-phenylpyrimido[1,2-*b*]indazol-4-yl)aniline (**4e**) displays the maximum fluorescence intensity at 518 nm and shows a fluorescence quantum yield of 0.068. The synthesized pyramido-fused indazoles have been evaluated for their free radical scavenging activity and compound **4f** showed good antioxidant activity.

## 1. Introduction

Multi-component reactions (MCRs) play an important role in combinatorial chemistry [1]. This method has the capability for producing desired small molecule drugs with various degrees of structural diversity [2]. When three or more than three substances or reactants are reacted simultaneously to produce defined target molecules these reactions are said to be MCRs. The products display features of all the inputs and thus afford greater possibilities for molecular diversity [3]. The cascade/domino/tandem process [4,5,6], involves subsequent transformations of functionalities produced in the previous step [7]. Normally, these reactions are easier to carry out by one pot synthesis than multi-step synthesis [8]. In addition, the isolation of an intermediates are not required in MCR conditions and they are perfect candidates for drug discovery and combinatorial synthesis [9]. The most desirable potential drug candidates are libraries of small organic molecules which have limitations as bioavailable therapeutics. We can built up very large libraries with in short time by using a small set of reactants [10]. Conversely, the significance of MCRs for discovery of novel drugs has been verified certified by substantial industries with academic aspects [11]. The studies have aimed to develop efficient MCR protocols for the generation of a series of heterocyclic compounds. MCRs such as the Strecker synthesis [12], Hantzsch synthesis [13], Biginelli synthesis [14], Mannich reaction [15], Kabachnik-Fields reaction [16], Bucherer-Bergs reaction [17], Gewald reaction [18], Willgerodt-Kindler reaction [19], Ugi reaction [20] and A3 coupling [21] are named reactions which include the carbonyl compounds as one of the reactants. The synthetic arylation via transition metal-free conditions [22] and synthesis of a series of unreported N-fused heterocycles [23,24] was reported by our group. Very recently, our research group proposed a metal catalysed method for the facile preparation of pyrimido-fused indazoles [25]. This is a metal catalysed A3 coupling reaction between aminoindazoles, alkynes and aldehydes, that further proceeds via 6-endo-dig cyclization focusing on the functionalization of pyrimido[1,2-*b*]indazole derivatives. As a continuation in our synthetic strategy, we report now a method to synthesize highly functionalized pyrimido-fused indazole derivatives in good to excellent yields via cascade/tandem/domino reactions under metal-free conditions. It offers simple reaction conditions, easy protocols and cheap starting materials.

## 2. Results and Discussion

**Scheme 1:**
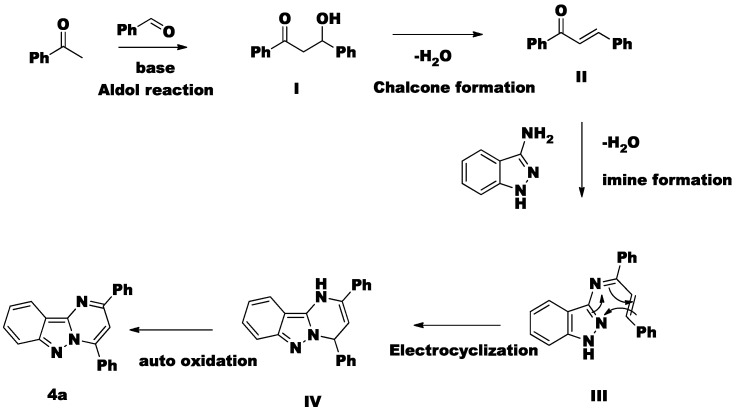
Plausible mechanism of the synthesis of compound **4a**.

The proposed protocol for the preparation of 2,4-diphenylpyrimido[1,2-*b*] indazole (**4a**) via the proposed metal-free cascade process is illustrated in Scheme 1. Initially, acetophenone **2a** and benzaldehyde **3a** were undergo an aldol reaction in the presence of base providing α,β-unsaturated carbonyl intermediate II. When intermediate II is coupled with an amine with the removal of one water molecule it gives an imine (intermediate III). Then intermediate III undergoes electrocyclization followed by auto-oxidation leading to the formation of **4a**.

**Scheme 2:**
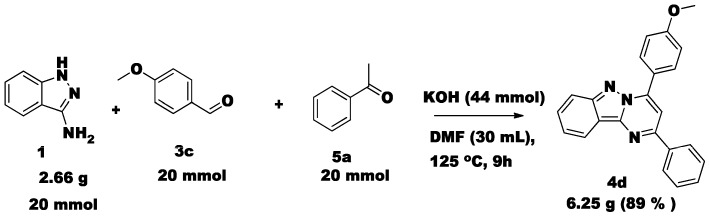
Compounds **4d** gram scale preparation.

**Figure 1:**
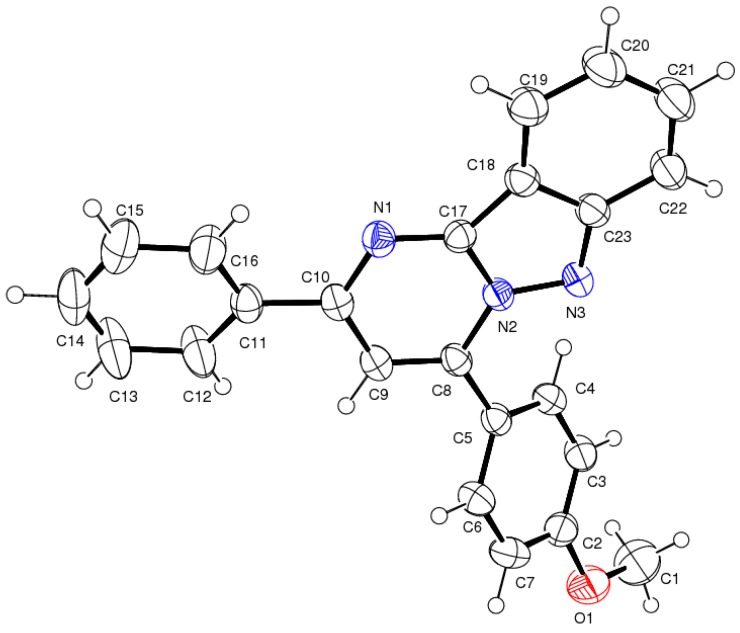
The ORTEP representation of compound **4d**.

Substituted aldehydes and acetophenones were transformed into the desired products without any difficulty, which clearly indicates that steric hindrance will not affect product formation. Unfortunately, nitro- and hydroxyl-substituted aromatic aldehydes failed to provide the target coupling products. We have investigated the gram scale preparation of compound **4d** (Scheme 2) from 20 mmol of 3-aminoindazole (**1**), 20 mmol of acetophenone (**5a**) and 20 mmol of 4-methoxy-benzaldehyde (**3d**) with good yield (89%). The synthesized compound **4d** was characterized by melting point, proton and carbon NMR and HRMS. Along with this data, we have confirmed the structure of **4d** by single crystal XRD data (CCDC 1456539). The ORTEP diagram is illustrated in Figure 1.

RSM is a statistical and mathematical method for empirical modelling and we have optimized a response (output variables) influenced by numerous independent variables (input variables) with a careful design of experiments. It can analyse the effect of multiple or combined variables in a minimal number of experiments and to evaluate the influences of experimental factors on the response by an accurate second order polynomial equation [26,27,28,29,30,31,32]. Aldehydes react with phenylacetylene and aminoindazole to give pyrimido[1,2-*b*]indazoles by C-2 subsitution in the presence of a metal (Scheme 1). Contrarily, aldehyde substitution occurs at the C-4 position in the absence of a metal catalyst. As part of our continuing effort, our research group has disclosed a simple and highly effective method for the preparation of *N*-fused pyrimido[1,2-*b*]indazoles **4**(**a**–**s**) via a domino process. RSM coupled with BBD was used for various conditions to fine-tune the working parameters. The selection level is based on teach variables (Table 1).

From the optimized A3 coupling method [25], we have observed that the aldehyde easily substituted at the C-2 position in the pyrimido-fused indazoles. In order to increase the scope of the products, we have developed another novel synthetic route to synthesize pyrimido-fused indazoles via metal-free one pot multi-MCRs. In this case, we found that the aldehydes are certainly substituted at C-4 position in the core structure. This cascade reaction proceeds under metal-free conditions with the reaction between aldehydes, ketones and amines. Our research group started performing reactions between 3-aminoindazole (**1**), acetophenone (**2a**) and benzaldehyde (**3a**) under neat conditions for 8 h, but unfortunately the expected compound was not identified (Table 2, entries 1 and 2). The conditions of reactions are optimized by varying organic solvent, organic bases and inorganic bases (Table 1, entries 3–15) but the expected target molecule was obtained between 16%–93%. After the various combinations of bases and solvents, we have found that the expected product 2,4-diphenylpyrimido[1,2-*b*]indazole **4a** in 56% yield was noted in KOH and EtOH combination at 80 °C for 8 h (Table 1, entry 9). In order to increase the yield further, we have tried with KOH base in various organic solvents like MeOH, DMSO, DMF, 1,4-dioxane, THF and acetonitrile. Finally, we have attained upto 93% yield in KOH/DMF combination at 120 °C for 8 h (Table 1, entry 12).

The BBD statistical design is presented in Table 3. We have utilized three variables such as base (**A**), temperature (**B**) and time (**C**) for the reactions without metal. By using multi-regression and backward eradication, the best-fitting models were determined.

**Figure 2:**
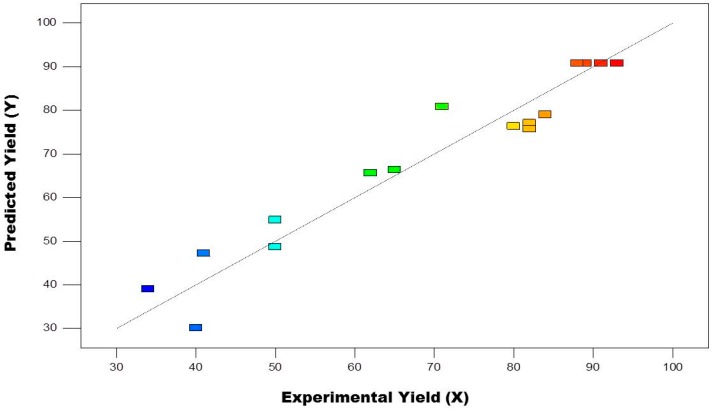
The predicted and actual isolated yield values.

Depending on the reaction phenomenon, we have found the yield range was 41%–93%. Due to the reaction performed by the above method the correlation between the process variables and the isolated yield (Y) with a quadratic polynomial model is represented in Equation (1):

Y = 90.80 + 8.25A + 2.88 B + 17.13C − 2.50 AB − 1.00 AC − 1.25 BC − 13.03 A − 9.28 B − 21.27C
(1)

where A, B and C represents the variables coded and Y represents isolated yield. Figure 2 depicts the respectable correlation linear between actual and predicted yields.

**Figure 3:**
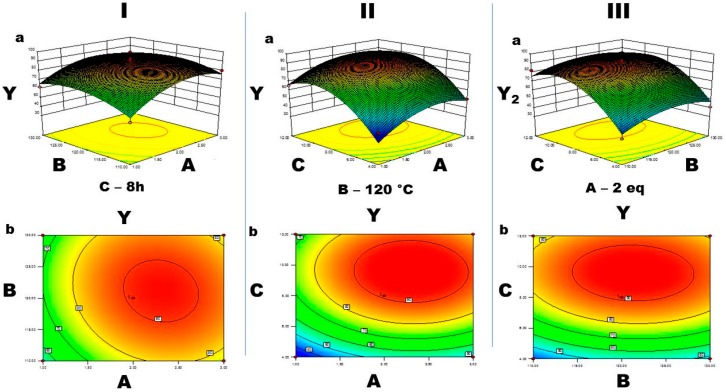
Contour plots for parameters combined yield for reaction conditions without metal. (**I**) Effect of A and B; (**II**) Effect of A and C; (**III**) Effect of B and C.

ANOVA results for response surface quadratic models are shown in Table 4. The F-value model is 11.57 and it suggests the model was significant. F value has a chance of 0.002% of being due to noise. For this model A, C, A2, B2, C2 are significant model terms. The values <0.1 represent that the model terms are non-significant. The lack of fit F value of 25.69 suggests that lack of fit is significant. The “Pred R-Squared” of 0.0372 is not as close to the “Adj R-Squared” of 0.8560 as one might usually expect; i.e., the difference is more than 0.002. All empirical models should be tested by doing confirmation runs. “Adeq Precision” noise to signal ratio measures and ratio <4 is desirable. The adequate signal with the ratio of 10.194 implies that we utilized the above model for space design navigation. Effect of interaction between base (A) and temperature of the reaction (B) time constant of reaction of 8 h (C) (Figure 3).

Plots of contour base (A) and temperature (B) (Figure 3IB) are circular, which result in lower a interaction between A and B. Plots of contour base (A) and time (C) (Figure 3IIB), temperature (B) and time (C) (Figure 3IIIC) are elliptical, which shows a good relation of interaction between A and C, and between B and C. The optimized condition for predicted and actual isolated yields is revealed in Table 5.

To further confirm the suitability for this model for identifying a higher yield, three confirmation runs were performed using the optimum parameters. The isolated yield results of compound **4a** were 93.0%, 92.4% and 93.7%. To validate the efficiency of the metal-free conditions, the choice of the reactants were explored under the optimized condition and the results are indicated in Table 6. A series of amines **1**(**a**–**d**), aryl aldehydes **3**(**a**–**k**) and acetophenones **2**(**a**–**f**) were subsequently converted into the corresponding pyrimido-fused indazoles **4**(**a**–**s**) in good to moderate yields.

The synthesized N-fused analogues **4**(**a**–**s**) were orange to red powders. The solvatochromism spectra of compound **4a** was studied with fifteen organic solvents (Figure S1). Ethyl acetate shows higher UV/Vis absorbance and we have recorded UV/Vis absorbances (Figure S2) and fluorescence emission spectra for all synthesized compounds **4**(**a**–**p**) in EtOAc at 10^−5^ M concentration.

**Figure 4:**
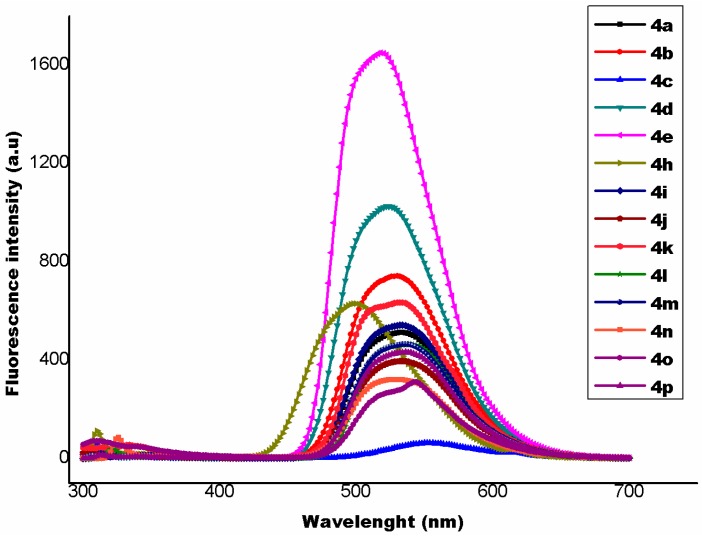
Fluorescence emission spectra the compounds **4**(**a**–**p**).

Compound **4e** displays a maximum fluorescence intensity at 518 nm (Figure 4). The fluorescence quantum yield [33] (ΦF) of the synthesized compounds **4**(**a**–**p**) have been calculated by using following Equation (2):

Φ_F_ = (Φ_R_ ×I_S_ × OD_R_ × ղ_s_)/(I_R_ × OD_S_ × ղ_R_) (2)

where Φ_R_ = fluorescence quantum yield of reference, I_S_ and I_R_ = integral area of reference and sample, respectively, OD_S_ and OD_R_ = excited absorbance of sample and reference respectively, ղ_s_ and ղ_R_ = refractive index of sample solvent and reference solvent respectively. We have used tryptophan as a standard for calculating the emission quantum yield.

The fluorescence quantum yield of the synthesized compounds **4**(**a**–**p**) ocurrs in the range of 0.010 to 0.068. The compound *N,N*-dimethyl-4-(2-phenylpyrimido[1,2-*b*]indazol-4-yl)aniline (**4e**) shows a fluorescence quantum yield of 0.068 (Table 7).

We have evaluated the free radical scavenging property of the synthesized pyrimido-fused indazoles at 0.001 mM and 0.002 mM and its IC_50_ values were measured. We have calculated the IC_50_ values and compound **4f** showed excellent antioxidant properties as 3.08 and compounds **4g** (8.91), **4n** (9.24), **4k** (8.59) and **4o** (5.60) showed significant antioxidant properties compared to ascorbic acid (4.50; Supplementary Materials Table S1).

## 3. Materials and Methods

### 3.1. General Information

All commercially available reagents were used without any further purification and the reactions were monitored by TLC. ^1^H- (400 MHz) and ^13^C-NMR (100 MHz) were obtained using an Avance 400 Mz spectrometer (Bruker, Oestliche Rheinbrueckenstr, Karlsruhe, Germany, Europe) in CDCl3 with TMS as an internal standard. Chemical shift values (δ) are expressed in parts per million (ppm). Abbreviations are as follows: s, singlet; d, doublet; t, triplet; m, multiplet. Melting points were measured on an Elchem microprocessor- based DT apparatus (Geninune Sientific Instruments, Chennai, Tamilnadu, India) using open capillary tubes and are corrected with benzoic acid. Mass spectra were obtained on a high resolution mass spectrometer (Bruker, Oestliche Rheinbrueckenstr, Karlsruhe, Germany, Europe). UV-vis spectra were obtained on a UV-2550 instrument (Shimadzu Corporation, Kyoto, Japan). The fluorescence spectra were obtained on a F-7000 FL spectrophotometer (HitachiPerkin-Elmer, Bengaluru, Karnataka, India).

### 3.2. General Procedure for the Synthesis of 2,4-Diphenylpyrimido[1,2-b]indazoles ***4**(**a**–**s**)* via Metal Free Conditions

To a mixture of 1*H*-indazol-3-amine (1 mmol), aldehyde (1 mmol) and acetophenone (1 mmol) in 5 mL of dimethylformamide potassium hydroxide (2.2 mmol) was added at room temperature. The reaction mixture was heated to 125 °C for 9 h. The progress of the reaction was monitored by TLC. After the completion of the reaction, the mixture was poured into crushed ice. The mixture was extracted by ethyl acetate and water. The organic layer was separated and dried over sodium sulphate and the solvent evaporated. The crude product was purified by column chromatography to afford the product as a solid.

### 3.3. Characterization Data

*4-(4-Isopropylphenyl)-2-phenylpyrimido[1,2-b]indazole* (**4b**); Brown solid; Isolated yield 86%; m.p.: 170–172 °C; ^1^H-NMR (CDCl_3_) δ 8.42 (d, *J* = 8.0 Hz, 1H), 8.28–8.26 (m, 2H), 8.18–8.16 (m, 2H), 7.96–7.85 (m, 1H), 7.86 (d, *J* = 8.0 Hz, 1H), 7.74 (s, 1H), 7.63–7.549 (m, 7H), 7.32–7.28 (m, 1H), 3.08–3.01 (m, 1H), 1.35 (d, *J* = 6.8 Hz, 6H); ^13^C-NMR (CDCl_3_) δ 23.8, 34.3, 108.4, 113.9, 116.5, 120.6, 121.2, 126.5, 127.0, 127.2, 128.0, 128.3, 128.5, 129.0, 129.2, 129.5, 130.0, 133.0, 137.4, 145.0, 145.4, 151.6, 152.2, 152.6; HRMS (EI): *m*/*z* calcd. for C_25_H_21_N_4_ 363.1735 found 363.1735.

*4-(4-Methoxyphenyl)-2-phenylpyrimido[1,2-b]indazole* (**4d**); Yellow solid; Isolated yield 90%; m.p.: 188–190 °C; ^1^H-NMR (CDCl_3_) δ 8.40 (d, *J* = 8.0 Hz, 1H), 8.26–8.22 (m, 4H), 7.84 (d, *J* = 8.8 Hz, 1H), 7.69 (s, 1H), 7.56–7.49 (m, 4H), 7.29–7.18 (m, 1H), 7.14–7.11 (m, 2H), 3.91 (s, 3H); ^13^C-NMR (CDCl_3_) δ 55.5, 107.8, 113.9, 114.2, 116.5, 120.5, 121.2, 123.9, 127.2, 129.0, 129.7, 130.0, 131.2, 137.5, 145.0, 145.1, 151.5, 152.5, 161.7; HRMS (EI): *m*/*z* calcd. for C_23_H_17_N_3_O 351.1372 found 351.1370.

*N,N-Dimethyl-4-(2-phenylpyrimido[1,2-b]indazol-4-yl)aniline* (**4e**); Brown solid; Isolated yield 85%; m.p.: 180–182 °C; ^1^H-NMR (CDCl_3_) δ 8.44 (d, *J* = 8.0 Hz, 1H), 8.33–8.30 (m, 4H), 7.99 (d, *J* = 8.4 Hz, 1H), 7.75 (s, 1H), 7.65–7.51 (m, 4H), 7.32–7.28 (m, 1H), 6.92 (d, *J* = 8.0 Hz, 2H), 3.13 (s, 6H); ^13^C-NMR (CDCl_3_) δ 40.1, 106.8, 111.6, 113.8, 116.4, 118.4, 120.1, 121.3, 127.2, 128.9, 129.5, 129.8, 130.8, 137.8, 145.2, 145.7, 151.5, 152.1, 152.5; HRMS (EI): *m*/*z* calcd. for C_24_H_20_N_4_ 364.1688 found 364.1688.

*4-(Naphthalen-1-yl)-2-phenylpyrimido[1,2-b]indazole* (**4j**); Brown solid; Isolated yield 85%; m.p.: 248–250 °C; ^1^H-NMR (CDCl_3_) δ 8.47 (d, *J* = 8.0 Hz, 1H), 8.30 (d, *J* = 7.6 Hz, 2H), 8.12 (d, *J* = 8.0 Hz, 1H), 8.00 (d, *J* = 8.0 Hz, 1H), 7.86 (d, *J* = 6.8 Hz, 1H), 7.79 (s, 1H), 7.76–7.65 (m, 2H), 7.57–7.31 (m, 8H); ^13^C-NMR (CDCl_3_) δ 110.8, 114.0, 116.8, 120.8, 121.1, 125.2, 125.4, 126.6, 127.1, 127.2, 128.2, 128.7, 129.1, 129.7, 129.8, 130.2, 130.7, 131.1, 133.7, 137.2, 144.7, 145.1, 151.7, 152.1; HRMS (EI): *m/z* calcd. for C_26_H_17_N_3_ 371.1422 found 371.1422.

*4-(Furan-2-yl)-2-phenylpyrimido[1,2-b]indazole* (**4k**); Brown solid; Isolated yield 78%; m.p.: 172–174 °C; ^1^H-NMR (CDCl_3_) δ 8.49–8.48 (m, 1H), 8.36 (d, *J* = 8.4 Hz, 1H), 8.25 (d, *J* = 8.0 Hz, 2H), 8.16 (s, 1H), 7.84 (d, *J* = 8.8 Hz, 1H), 7.70 (s, 1H), 7.83–7.79 (m, 4H), 7.25 (t, *J* = 8.0 Hz, 1H), 6.73-6.72 (m, 1H); ^13^C-NMR (CDCl_3_) δ 103.4, 113.2, 113.8, 116.4, 119.7, 120.7, 121.3, 127.2, 129.0, 130.0, 134.4, 137.6, 144.7, 145.0, 145.4, 151.8; HRMS (EI): *m/z* calcd. for C_20_H_13_N_3_O 311.1059 found 311.1058.

*2-Phenyl-4-(thiophen-2-yl)pyrimido[1,2-b]indazole* (**4l**); Brown solid; Isolated yield 86%; m.p.: 190–192 °C; ^1^H-NMR (CDCl_3_) δ 8.59–8.58 (m, 1H), 8.42 (d, *J* = 7.6 Hz, 1H), 8.28 (d, *J* = 8.0 Hz, 2H), 8.09 (s, 1H), 7.94 (d, *J* = 8.8 Hz, 1H), 7.79 (d, *J* = 5.2 Hz, 1H), 7.68–7.52 (m, 4H), 7.36–7.30 (m, 2H); ^13^C-NMR (CDCl_3_) δ 104.8, 113.9, 116.5, 120.8, 121.4, 127.2, 127.7, 129.0, 130.0, 130.1, 131.3, 132.1, 132.2, 137.5, 138.5, 145.2, 151.4, 152.0; HRMS (EI): *m/z* calcd. for C_20_H_13_N_3_S 327.0830 found 327.0830.

### 3.4. Experimental Design and Mathematical Model

An experimental design was established for the series of parameters used for the synthesis of 2,4-diphenylpyrimido [1,2-*b*] indazoles by two reaction methods such as metal mediated and metal-free conditions. The model was built by Response Surface Methodology (RSM) with the Design-Expert Version 9.0.5.1 software (Stat-Ease, Inc., Minneapolis, MN, USA). Levels of selection for each variable were based on the results of the preliminary studies. The three components for each reaction method, such as the catalyst loading (A1), reaction temperature (B1) and response time (C1) were utilized for metal mediated reaction. For metal free reaction, we have used base equivalent (**A_2_**), reaction temperature (**B_2_**) and reaction time (**C_2_**) as three factors. The actual isolated yields **Y_1_** and **Y_2_** were chosen to be the target or response parameter as dependent variables. The values **X_1_** and **X_2_** are the predicted isolated yields. Seventeen sets of experiments were performed for each both reaction methods according to Box-Behnken experimental design (BBD). The variables were tested at the three levels by associating negative sign (−1) for lower level, Zero (0) indicating the core value and plus signs (+1) for higher stages (Table 1). The experimental design matrix and their effects are presented in Table S1. The quadratic polynomial equation recommended by RSM was used to predict the optimal value and examine the interaction between the response of experimental design (actual yield) and the variables (process parameters). The general form of quadratic polynomial was as follows:

***Y= β_0_ + β_1_X_1_ + β_2_X_2_ + β_3_X_3_ + β_11_X_1_^2^ + β_22_X_2_^2^ + β_33_X_3_^2^ + β_12_X_1_X_2_ + β_13_X_1_X_2_+ β_23_X_2_X_3_*** (3)

where ***β*_0_** is constant coefficient of the models. The regression coefficients (***β_1_***, ***β_2_*** and ***β_3_***), (***β_11_***, ***β_22_*** and ***β_33_***) and (***β_12_***, ***β_13_*** and ***β_23_***) respectively represent linear, quadratic and interaction effects of the model estimated by multiple regression analysis.

### 3.5. Antioxidant Activity

The free radical scavenging activity of the pyrimido-fused indazoles was determined according to the method using our earlier reports [34].

## 4. Conclusions

A metal-free MCR has been developed to synthesize a series of pyrimido-fused indazoles via cascade/tandem transformation. This KOH/DMF mediated three component cascade type transformation offers good to moderate yields. It offers good functional group tolerance. The RSM coupled with BBD results displayed the significance of the quadratic model and offered fine-tuned conditions for the preparation of pyrimido[1,2-*b*]indazole derivatives. The synthesized analogues showed better fluorescence properties. The synthesized pyrimido-fused indazoles were evaluated as good free radical scavengers.

## Figures and Tables

**Table 1:** A selection of levels and variables used for BBD.

Reaction	Variables	Code	Units	Levels		
				−1	0	+1
Metal free cascade Reaction	Base used (equivalent)	A	mg	1	2	3
	Reaction temperature	B	°C	110	120	130
	Reaction time	C	h	4	8	12

**Table 2:**
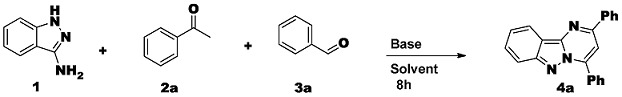
Optimization of cascade reaction to synthesis compound 2,4-diphenylpyrimido[1,2-*b*]indazole (**4a**) via metal free conditions ^a^.

Entry	Base	Solvent	Temp (°C)	Yield ^b^ (%)
1	-	-	100	NR ^c^
2	-	EtOH	80	NR ^c^
3	Triethylamine	EtOH	80	NR ^c^
4	Na_2_CO_3_	EtOH	80	Traces
5	Piperidine	EtOH	80	NR ^c^
6	NaO^t^Bu	*t*-BuOH	100	40
7	KO^t^Bu	*t*-BuOH	100	30
8	NaOH	EtOH	80	50
9	KOH	EtOH	80	56
10	KOH	MeOH	70	40
11	KOH	DMSO	120	45
**12**	**KOH**	**DMF**	**120**	**93**
13	KOH	1,4-Dioxane	80	25
14	KOH	THF	80	16
15	KOH	Acetonitrile	80	16

^a^ Reactions proceed with 1 mmol of **1**, **2a** and **3a** in 5 mL of solvent. Optimized condition are denoted in bold letters there are none. The reactions were carried out at various temperatures for 8 h. ^b^ Isolated yield and ^c^ NR-No reaction. Letters refer to wrong things.

**Table 3:** BBD matrix response and its design ^a^.

Run	Metal Free Condition				
	A	B	C	Y (%)	X (%)
	2	120	8	93.01	90.80
	2	120	8	89.00	90.80
	1	120	4	40.33	30.12
	1	130	8	62.03	65.62
	3	130	8	82.09	77.12
	2	110	4	34.12	39.00
	3	110	8	80.31	76.37
	3	120	4	50.41	48.62
	2	120	8	91.01	90.80
	3	120	12	71.00	80.88
	1	110	8	50.21	54.87
	2	110	12	82.07	75.75
	2	130	4	41.09	47.25
	1	120	12	65.21	66.37
	2	120	8	88.44	90.80
	2	130	12	84.51	79.00
	2	120	8	93.01	90.80

^a^ Y—Experimental Yield, X—Predicted Yield.

**Table 4:** Quadratic model by ANOVA for response surface ^a^.

Source	Sum of Squares	DF	Mean Square	F Value	*p*-Value Prob > F	
Model	6271.98	9	696.89	11.57	0.0020	S
A	544.50	1	544.50	9.04	0.0197	
B	66.13	1	66.13	1.10	0.3295	
C	2346.12	1	2346.12	38.96	0.0004	
AB	25.00	1	25.00	0.42	0.5399	
AC	4.00	1	4.00	0.066	0.8040	
BC	6.25	1	6.25	0.10	0.7567	
A	714.32	1	714.32	11.86	0.0108	
B	362.21	1	362.21	6.01	0.0439	
C	1905.79	1	1905.79	31.65	0.0008	
Residual	421.55	7	60.22			
Lack of Fit	400.75	3	133.58	25.69	0.0045	S
Pure Error	20.80	4	5.20			
Correlation Total	6693.53	16				

^a^ Where S—Significant, DF—Degree of freedom.

**Table 5:** Model validation for metal free reaction.

Parameter	Base Equivalent (A)	Reaction Temperature °C (B)	Reaction Time (min) (C)	% Yield
Predicted	2.23	125.3	9.17	93.5
Experimental	2.0	120.0	8.0	93.0

**Table 6:** Syntheses of N-fused pyrimidine derivatives **4**(**a**–**p**).

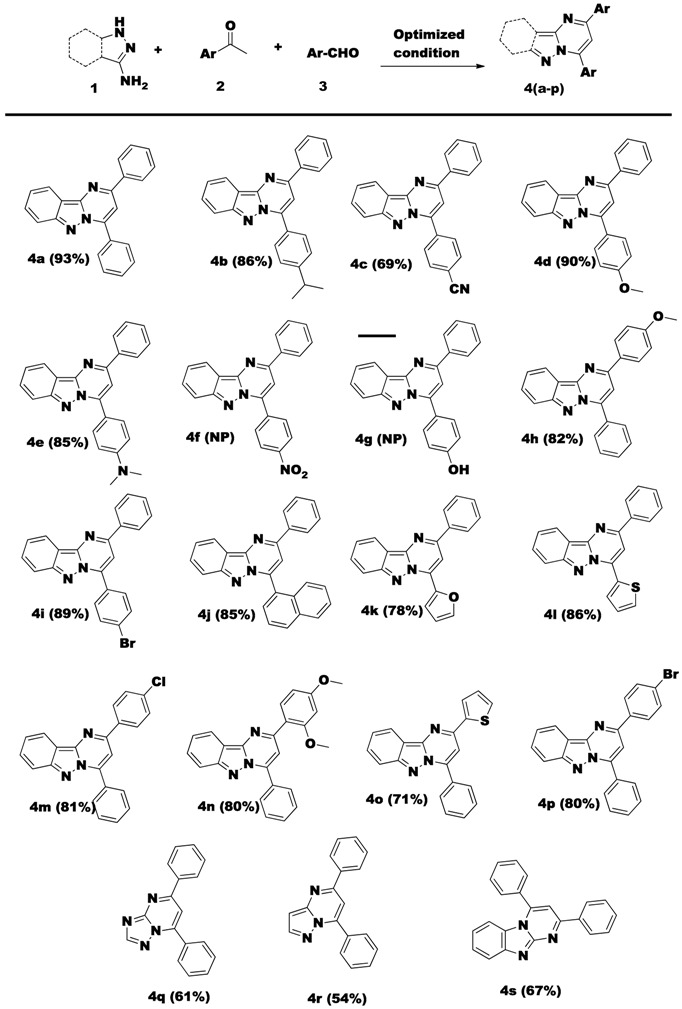

**Table 7:** The optical properties of the synthesized pyrimido[1,2-*b*]indazolederivatives **4**(**a**–**p**).

Entry	Λmax (abs, nm)	Λmax (em, nm)	Stokes Shift (nm)	OD	I	Φ_F_
Tryptophan [32]	280	355	75	0.384	158,517	0.130
**4a**	306	533	227	0.774	46,182	0.019
**4b**	306	530	224	0.957	90,759	0.031
**4c**	308	553	245	1.064	31,279	0.010
	270 (Sh)	553	283	0.838	31,279	0.012
**4d**	310	524	214	1.505	115,328	0.025
**4e**	300	518	218	0.870	165,016	0.062
	344 (Sh)	518	174	0.788	165,016	0.068
**4h**	310	524	214	1.505	115,328	0.025
**4i**	306	534	228	1.883	73,195	0.013
	270	534	264	1.251	73,195	0.019
**4j**	300	534	234	1.140	61,312	0.017
**4k**	314	532	218	1.950	83,407	0.014
**4l**	320	540	220	2.070	65,088	0.010
**4m**	308	535	227	1.794	70,535	0.013
**4n**	272	530	258	0.449	54,760	0.040
	322 (Sh)	530	208	0.439	54,760	0.041
**4o**	270	543	273	1.761	29,458	0.005
**4p**	306	499	193	1.176	59,599	0.016
	268 (Sh)	499	231	0.827	59,599	0.023

Sh = shoulder; abs = absorbance; em = emission; OD = excited absorbance; I = integral area; Φ_F_ = Fluorescence quantum yield.

## Supplementary Materials

Supplementary materials can be accessed at: http://www.mdpi.com/1420-3049/21/11/1571/s1.
