# Supplementary material for: A Metal-Free Regioselective Multicomponent Approach for the Synthesis of Free Radical Scavenging Pyrimido-Fused Indazoles and Their Fluorescence Studies
Source: Molecules. 2016 Nov 18;21(11):1571. doi: 10.3390/molecules21111571 (PMC6273232; doi:10.3390/molecules21111571)
Supplement: Supplementary file 1 [file molecules-21-01571-s001.pdf]

Supplementary Materials: Metal Free Regioselective Multicomponent Approach for the Synthesis of Free Radical Scavenging Pyrimido Fused Indazoles and Its Fluorescence Studies

Jeyakannu Palaniraja, Selvaraj Mohana Roopan, G Mokesh Rayalu, Naif Abdullah Al-Dhabi and MariadhasValan Arasu

1. Experimental Section

All commercially available reagents were used without any further purification and the reactions were monitored by TLC. ^1H- (400 MHz) and ^{13}C-NMR (100 MHz) were obtained using an Avance 400 Mz spectrometer (Bruker, Oestliche Rheinbrueckenstr, Karlsruhe, Germany, Europe) in CDCl3 with TMS as an internal standard. Chemical shift values (δ) are expressed in parts per million (ppm). Abbreviations are as follows: s, singlet; d, doublet; t, triplet; m, multiplet. Melting points were measured on an Elchem microprocessor- based DT apparatus (Geninune Sientific Instruments, Chennai, Tamilnadu, India) using open capillary tubes and are corrected with benzoic acid. Mass spectra were obtained on a high resolution mass spectrometer (Bruker, Oestliche Rheinbrueckenstr, Karlsruhe, Germany, Europe). UV-vis spectra were obtained on a UV-2550 instrument (Shimadzu Corporation, Kyoto, Japan). The fluorescence spectra were obtained on a F-7000 FL spectrophotometer (HitachiPerkin-Elmer, Bengaluru, Karnataka, India).

2. Experimental Design & Mathematical Model

An experimental design for the series of parameters used for the synthesis of 2,4-diphenylpyrimido [1,2-*b*] indazole by two reaction methods such as metal mediated and metal free conditions. The model was built by Response Surface Methodology (RSM) with the Design-Expert Version 9.0.5.1 (State-Ease, Inc., Minneapolis, Suite 480, MN 55413-2726, USA). Levels of selection for each variable based on the results of the preliminary studies. The three components for each reaction method, such as the catalyst loading (A1), reaction temperature (B1) and response time (C1) were utilized for metal mediated reaction. For metal free reaction, we have used base equivalent ($\mathbf{A}_2$), reaction temperature ($\mathbf{B}_2$) and reaction time ($\mathbf{C}_2$) as three factors. The actual isolated yields $\mathbf{Y}_1$ and $\mathbf{Y}_2$ were chosen to be the target or response parameter as dependent variables. The $\mathbf{X}_1$ and $\mathbf{X}_2$ is the predicted isolated yields. Seventeen sets of experiments were performed for each both reaction methods according to Box-Behnken experimental design (BBD). The variables were tested at the three levels by associating negative sign (−1) for lower level, Zero (0) indicating the core value and plus signs (+1) for higher stages (Table 1). The experimental design matrix and their effects are presented in Table S1.The quadratic polynomial equation recommended by RSM was used to predict the optimal value and examine the interaction between the response of experimental design (actual yield) and the variables (process parameters). The general form of quadratic polynomial was as follows

$$Y = \beta_0 + \beta_1 X_1 + \beta_2 X_2 + \beta_3 X_3 + \beta_{11} X_1^2 + \beta_{22} X_2^2 + \beta_{33} X_3^2 + \beta_{12} X_1 X_2 + \beta_{13} X_1 X_2 + \beta_{23} X_2 X_3 \quad (1)$$

Where β_0 is constant coefficient of the models. The regression coefficients (β_1, β_2 and β_3), (β_{11}, β_{22} and β_{33}) and (β_{12}, β_{13} and β_{23}) respectively represent linear, quadratic and interaction effects of the model estimated by multiple regression analysis.

Melting point comparison of synthesized compounds with reported compounds:

The reported compounds are confirmed by its melting points.

Entry	Structure	Melting Point (°C)	
		Obtained	Reported
4a	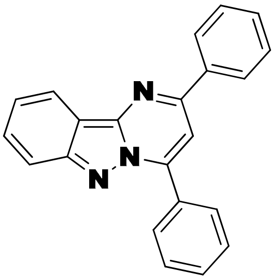	155–156	154–156 [25]
4c	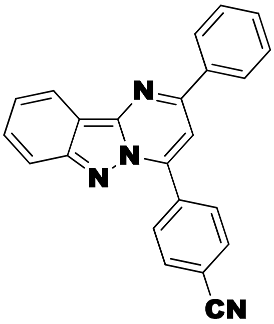	232–234	232–234 [25]
4h	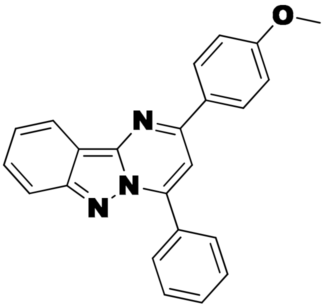	171–173	170–172 [25]
4i	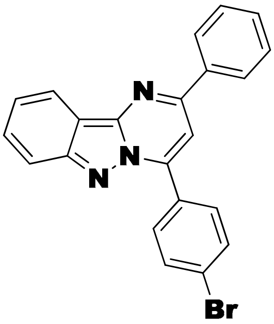	170–172	170–172 [25]
4m	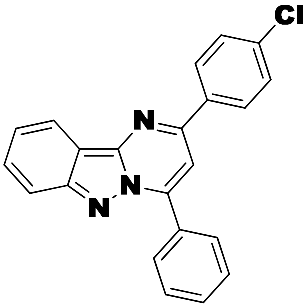	203–204	202–204 [25]

4n	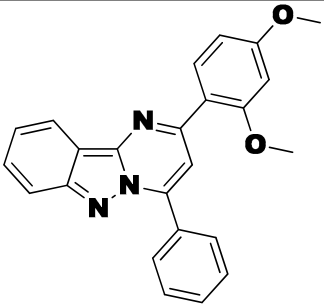	161–163	160–162 [25]
4o	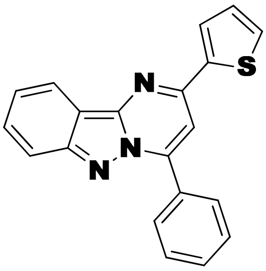	202–204	202–204 [25]
4p	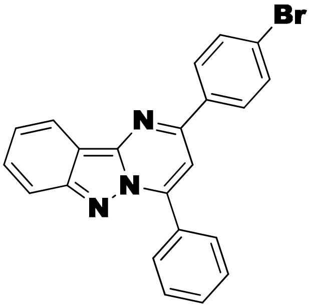	168–170	168–170 [25]
4q	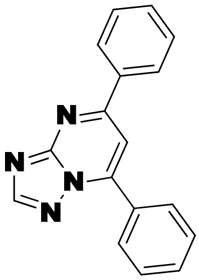	160–161	160–161 [25]
4r	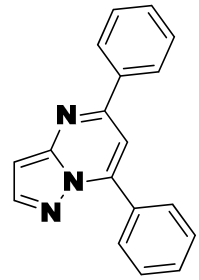	84–85	84–85[25]
4s	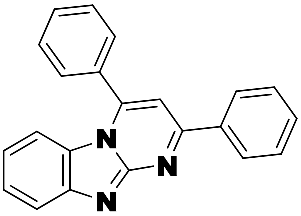	277–278	276–277 [25]

[25] represents reference article 25.

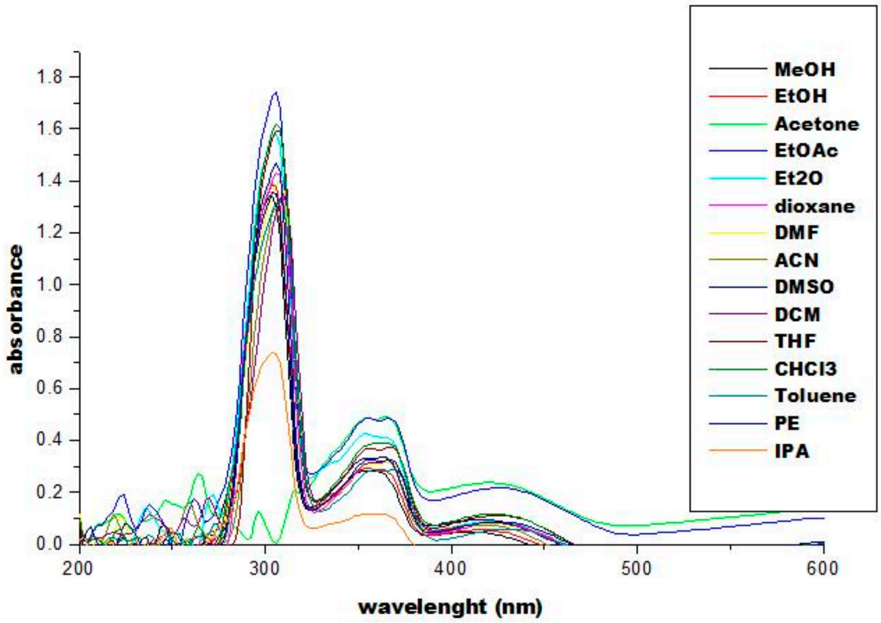

Figure S1. The solvatochromism spectra of the compound **4a**.

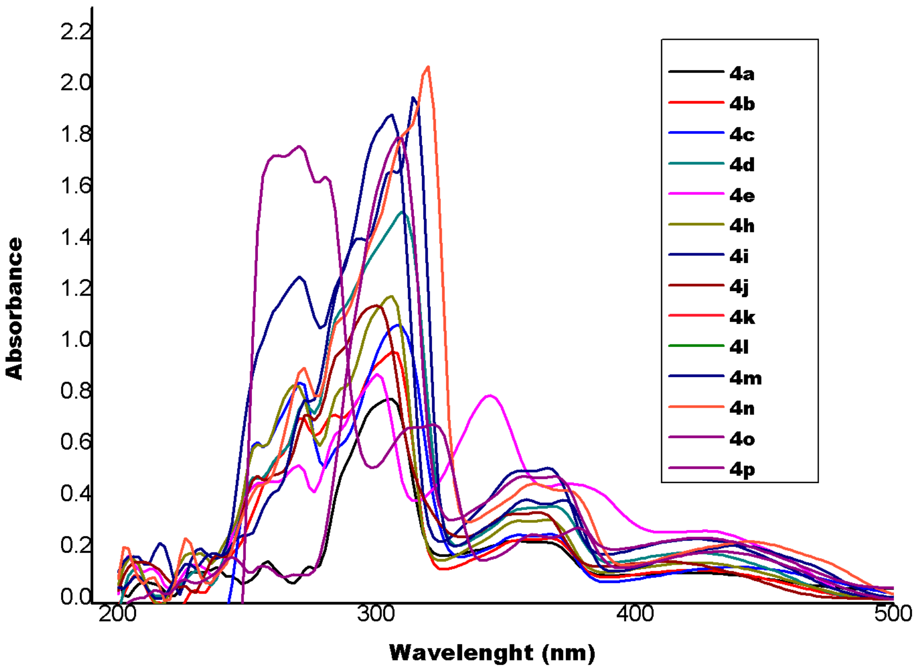

Figure S2. UV/Vis absorbance spectra of the pyrimido[1,2-*b*]indazoles **4**(**a**–**p**) in EtOAc.

2.1. Spectral Characterization of the Synthesized Compounds:

2.1.1. 4-(4-Isopropylphenyl)-2-phenylpyrimido[1,2-*b*]indazole (**4b**)

Brown solid; Isolated yield −86%; m.p.: 170–172 °C; ^{1}H-NMR (400 MHz, $CDCl_3$) δ 8.42 (d, *J* = 8.0 Hz, 1H), 8.28–8.26 (m, 2H), 8.18–8.16 (m, 2H), 7.96–7.85 (m, 1H), 7.86 (d, *J* = 8.0 Hz, 1H), 7.74 (s, 1H), 7.63–7.549 (m, 7H), 7.32–7.28 (m, 1H), 3.08–3.01 (m, 1H), 1.35 (d, *J* = 6.8 Hz, 6H); ^{13}C-NMR (100 MHz, $CDCl_3$) δ 23.8, 34.3, 108.4, 113.9, 116.5, 120.6, 121.2, 126.5, 127.0, 127.2, 128.0, 128.3, 128.5, 129.0, 129.2, 129.5, 130.0, 133.0, 137.4, 145.0, 145.4, 151.6, 152.2, 152.6; HRMS (EI): *m*/*z* calcd. for $C_{25}H_{21}N_4$ 363.1735 found 363.1735.

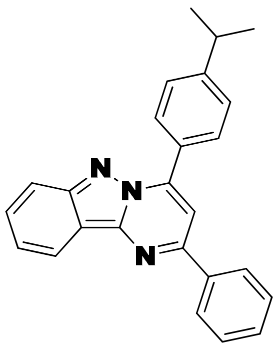

2.1.2. 4-(4-Methoxyphenyl)-2-phenylpyrimido[1,2-*b*]indazole (**4d**)

Yellow solid; Isolated yield −90%; m.p.: 188–190 °C; ^{1}H-NMR (400 MHz, $CDCl_3$) δ 8.40 (d, *J* = 8.0 Hz, 1H), 8.26–8.22 (m, 4H), 7.84 (d, *J* = 8.8 Hz, 1H), 7.69 (s, 1H), 7.56–7.49 (m, 4H), 7.29–7.18 (m, 1H), 7.14–7.11 (m, 2H), 3.91 (s, 3H); ^{13}C-NMR (100 MHz, $CDCl_3$) δ 55.5, 107.8, 113.9, 114.2, 116.5, 120.5, 121.2, 123.9, 127.2, 129.0, 129.7, 130.0, 131.2, 137.5, 145.0, 145.1, 151.5, 152.5, 161.7; HRMS(EI): *m*/*z* calcd. for $C_{23}H_{17}N_3O$ 351.1372 found 351.1370.

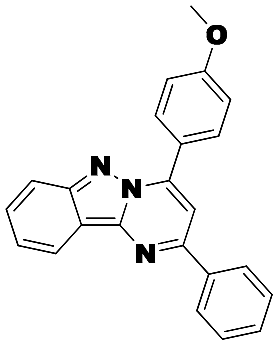

2.1.3. N,*N*-Dimethyl-4-(2-phenylpyrimido[1,2-b]indazol-4-yl)aniline (**4e**)

Brown solid; Isolated yield −85%; m.p.: 180–182 °C; ^{1}H-NMR (400 MHz, $CDCl_3$) δ 8.44 (d, *J* = 8.0 Hz, 1H), 8.33–8.30 (m, 4H), 7.99 (d, *J* = 8.4 Hz, 1H), 7.75 (s, 1H), 7.65–7.51 (m, 4H), 7.32–7.28 (m, 1H), 6.92 (d, *J* = 8.0 Hz, 2H), 3.13 (s, 6H); ^{13}C-NMR (100 MHz, $CDCl_3$) δ 40.1, 106.8, 111.6, 113.8, 116.4, 118.4, 120.1, 121.3, 127.2, 128.9, 129.5, 129.8, 130.8, 137.8, 145.2, 145.7, 151.5, 152.1, 152.5; HRMS(EI): *m*/*z* calcd. for $C_{24}H_{20}N_4$ 364.1688 found 364.1688.

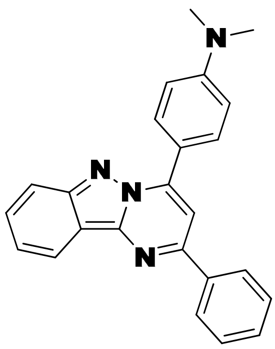

2.1.4. 4-(Naphthalen-1-yl)-2-phenylpyrimido[1,2-b]indazole (**4j**)

Brown solid; Isolated yield −85%; m.p.: 248–250 °C; ^{1}H-NMR (400 MHz, $CDCl_3$) δ 8.47 (d, *J* = 8.0 Hz, 1H), 8.30 (d, *J* = 7.6 Hz, 2H), 8.12 (d, *J* = 8.0 Hz, 1H), 8.00 (d, *J* = 8.0 Hz, 1H), 7.86 (d, *J* = 6.8 Hz, 1H), 7.79 (s, 1H), 7.76–7.65 (m, 2H), 7.57–7.31 (m, 8H); ^{13}C-NMR (100 MHz, $CDCl_3$) δ 110.8, 114.0, 116.8, 120.8, 121.1, 125.2, 125.4, 126.6, 127.1, 127.2, 128.2, 128.7, 129.1, 129.7, 129.8, 130.2, 130.7, 131.1, 133.7, 137.2, 144.7, 145.1, 151.7, 152.1; HRMS(EI): *m*/*z* calcd. for $C_{26}H_{17}N_3$ 371.1422 found 371.1422.

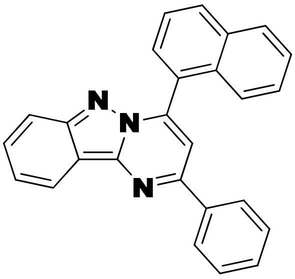

2.1.5. 4-(Furan-2-yl)-2-phenylpyrimido[1,2-b]indazole (**4k**)

Brown solid; Isolated yield −78%; m.p.: 172–174 °C; ^{1}H-NMR (400 MHz, $CDCl_3$) δ 8.49–8.48 (m, 1H), 8.36 (d, *J* = 8.4 Hz, 1H), 8.25 (d, *J* = 8.0 Hz, 2H), 8.16 (s, 1H), 7.84 (d, *J* = 8.8 Hz, 1H), 7.70 (s, 1H), 7.83–7.79 (m, 4H), 7.25 (t, *J* = 8.0 Hz, 1H), 6.73–6.72 (m, 1H); ^{13}C-NMR (100 MHz, $CDCl_3$) δ 103.4, 113.2, 113.8, 116.4, 119.7, 120.7, 121.3, 127.2, 129.0, 130.0, 134.4, 137.6, 144.7, 145.0, 145.4, 151.8; HRMS(EI): *m*/*z* calcd. for $C_{20}H_{13}N_3O$ 311.1059 found 311.1058.

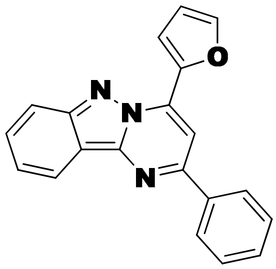

2.1.6. 2-Phenyl-4-(thiophen-2-yl)pyrimido[1,2-b]indazole (**4l**)

Brown solid; Isolated yield −86%; m.p.: 190–192 °C; ^{1}H-NMR (400 MHz, $CDCl_3$) δ 8.59–8.58 (m, 1H), 8.42 (d, *J* = 7.6 Hz, 1H), 8.28 (d, *J* = 8.0 Hz, 2H), 8.09 (s, 1H), 7.94 (d, *J* = 8.8 Hz, 1H), 7.79 (d, *J* = 5.2 Hz, 1H), 7.68–7.52 (m, 4H), 7.36–7.30 (m, 2H); ^{13}C-NMR (100 MHz, $CDCl_3$) δ 104.8, 113.9, 116.5, 120.8, 121.4, 127.2, 127.7, 129.0, 130.0, 130.1, 131.3, 132.1, 132.2, 137.5, 138.5, 145.2, 151.4, 152.0; HRMS(EI): *m*/*z* calcd. for $C_{20}H_{13}N_3S$ 327.0830 found 327.0830.

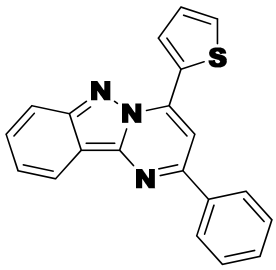

2.2. Copies of ¹H-NMR, ¹³C-NMR and HRMS

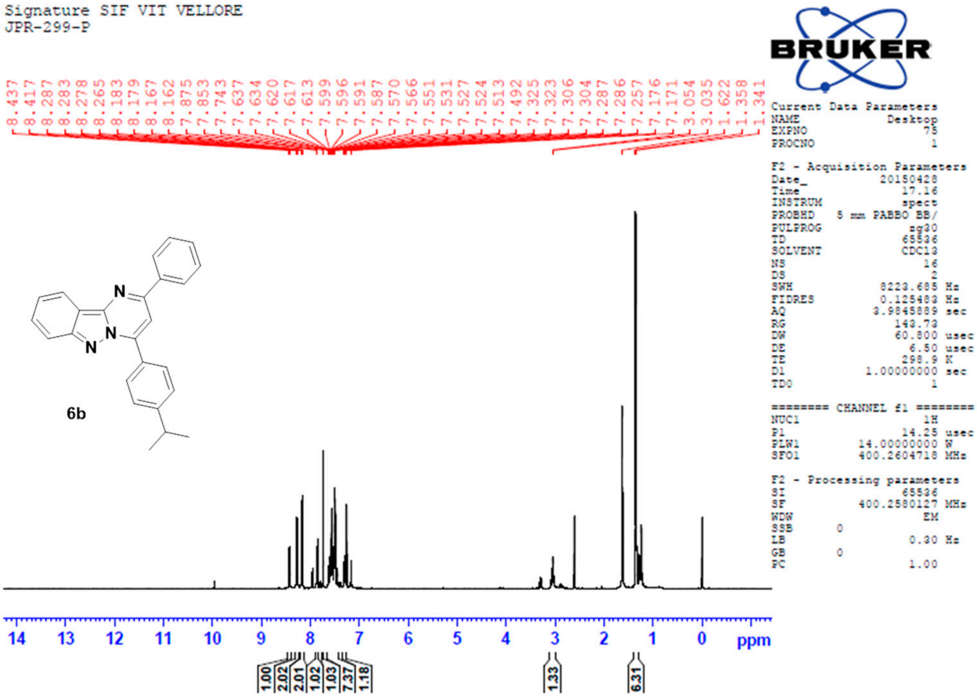

Figure S3. ¹H-NMR Spectrum of Compound (**4b**).

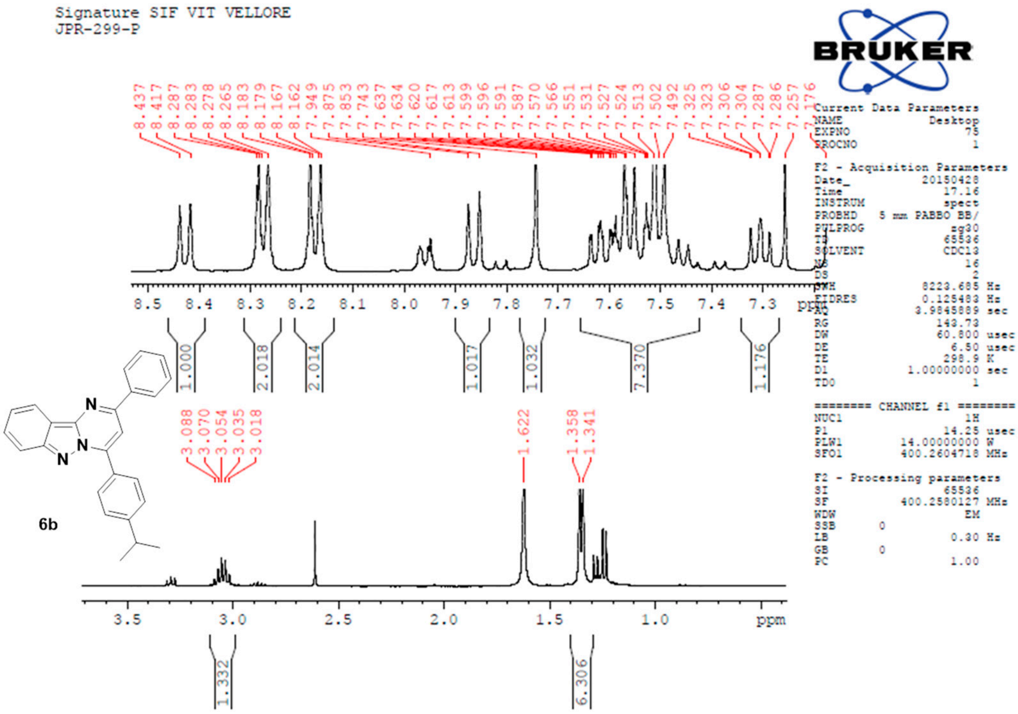

Figure S4. Expanded ¹H-NMR spectrum of compound (**4b**).

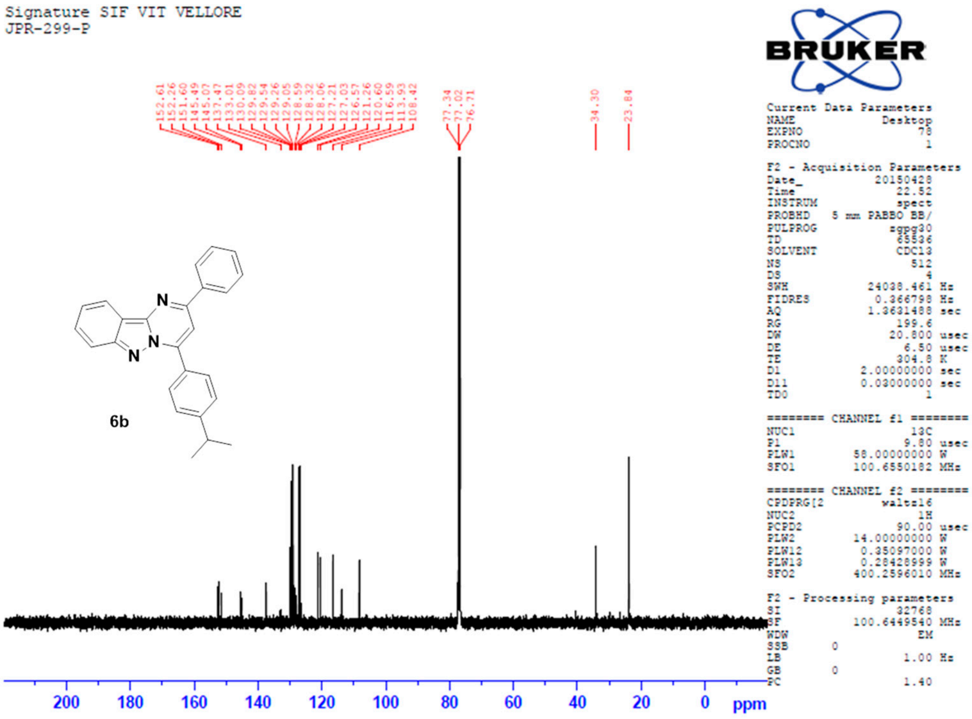

Figure S5. ^{13}C-NMR spectrum of compound (**4b**).

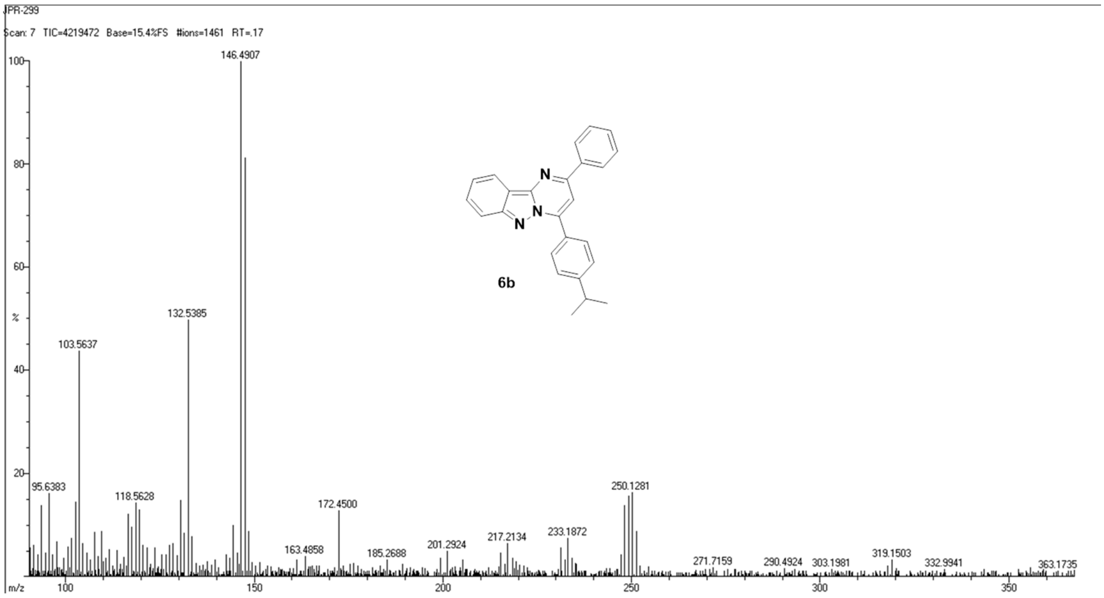

Figure S6. HRMS spectrum of compound (**4b**).

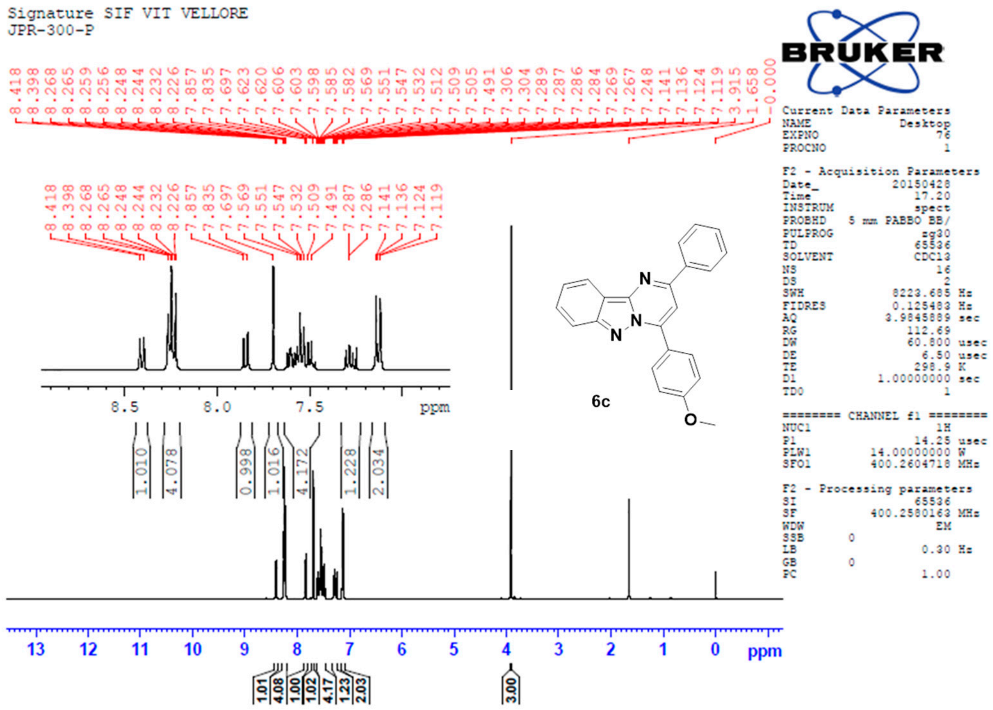

Figure S7. ¹H-NMR spectrum of compound (**4b**).

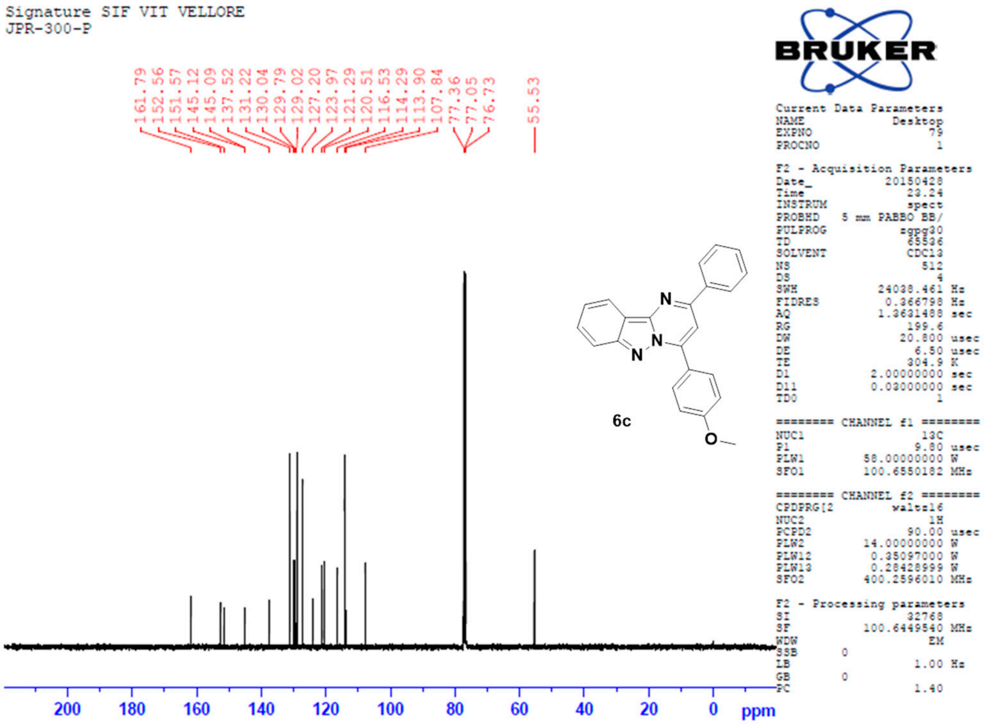

Figure S8. ¹³C-NMR spectrum of compound (**4b**).

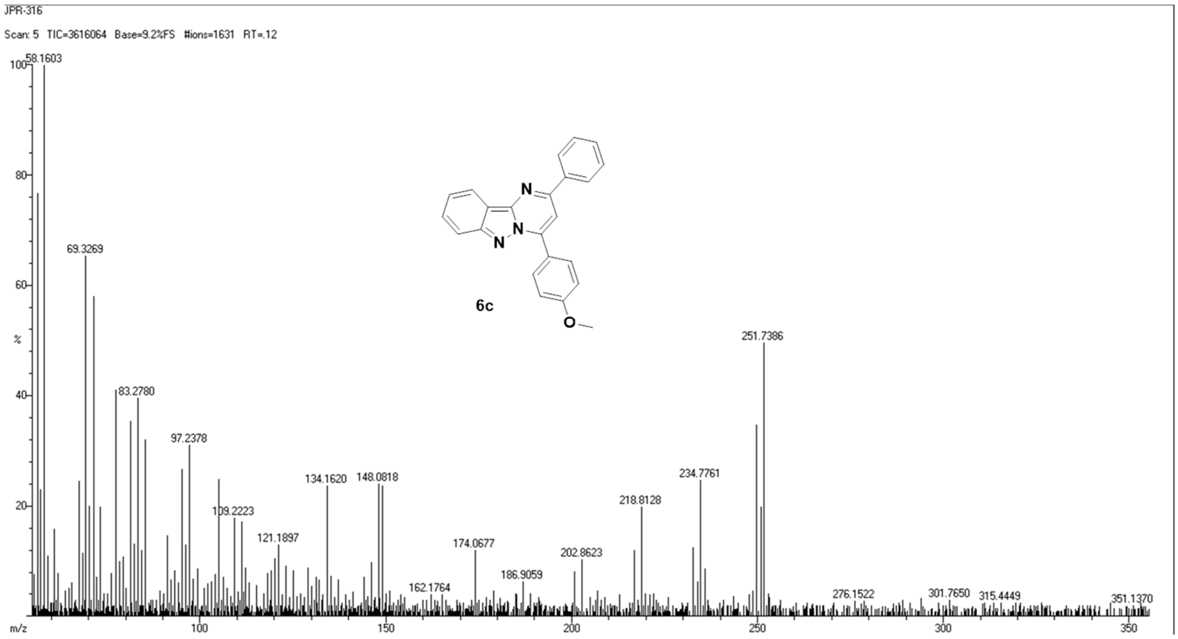

Figure S9. HRMS spectrum of compound (**4b**).

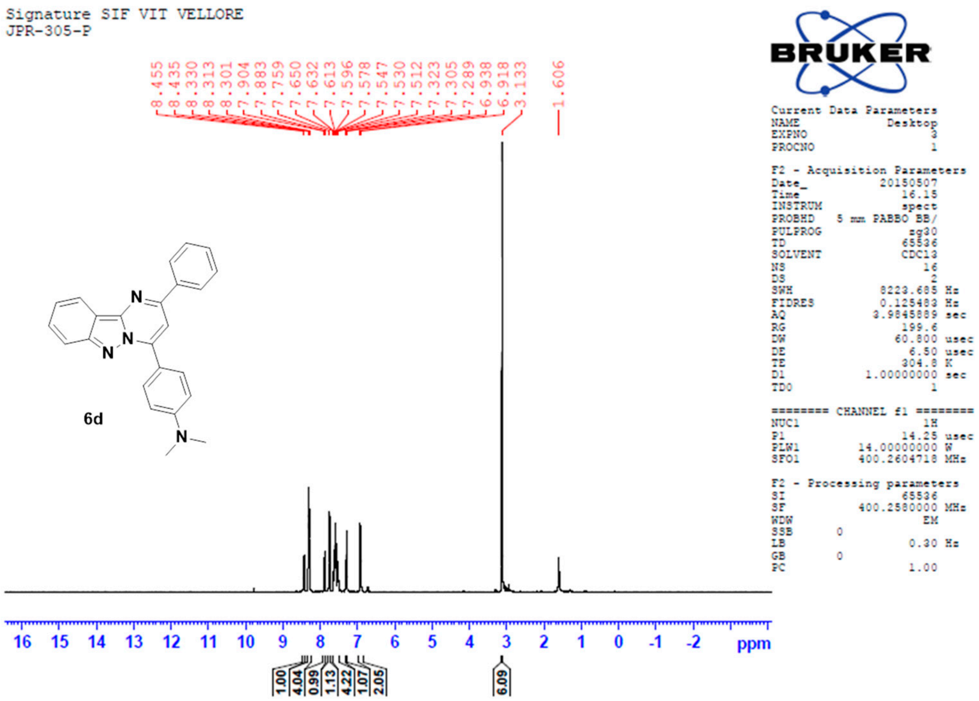

Figure S10. ^{1}H-NMR spectrum of compound (**4e**).

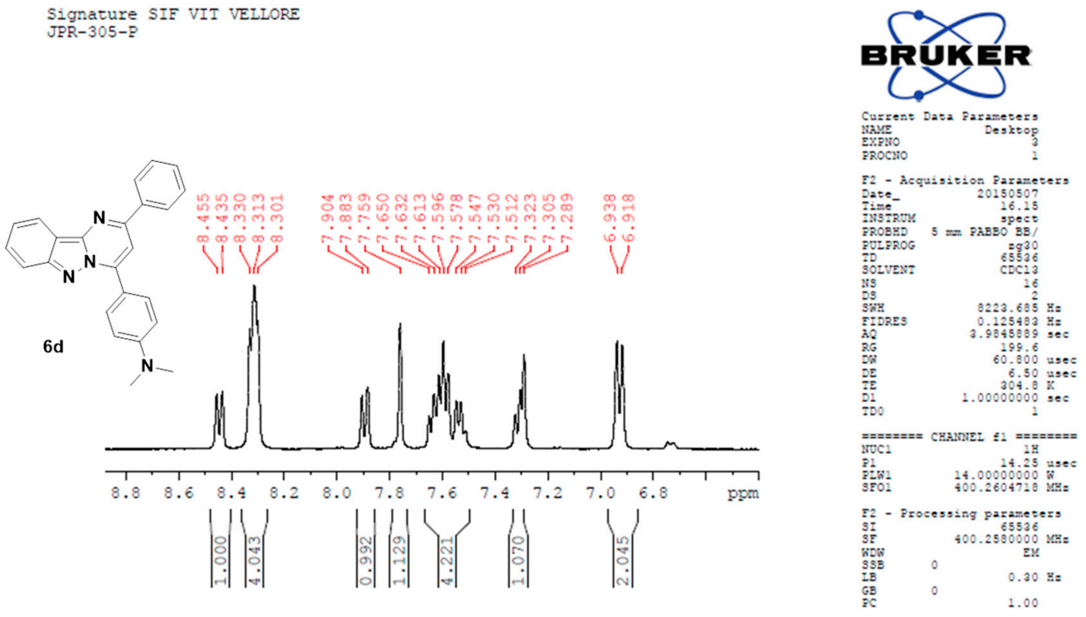

Figure S11. Expanded ^{1}H-NMR spectrum of compound (**4e**).

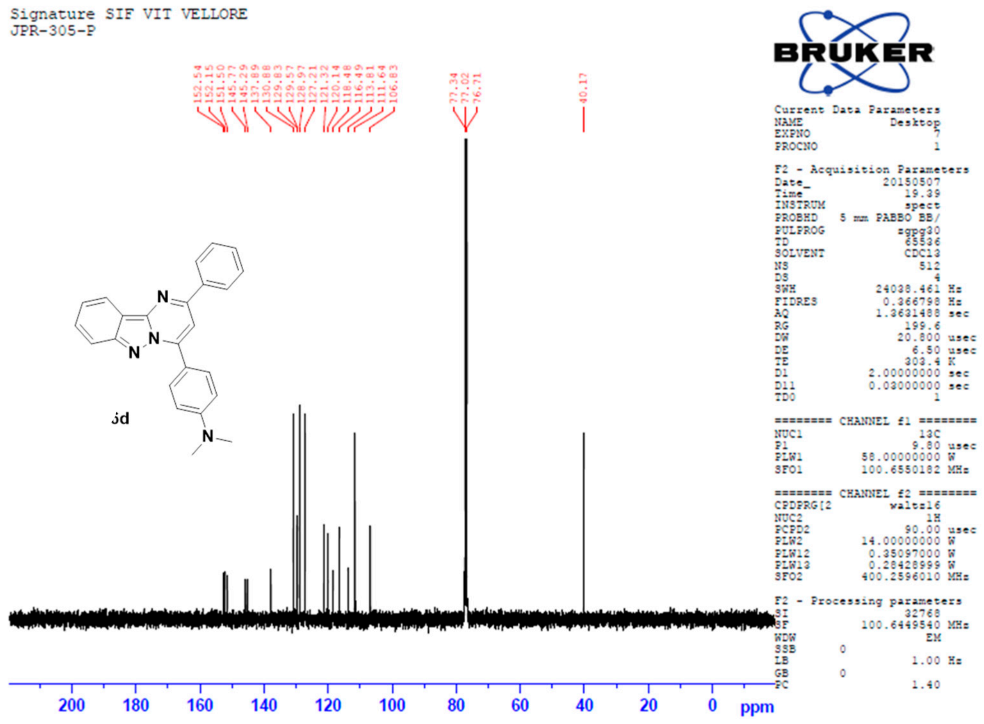

Figure S12. ^{13}C-NMR spectrum of compound (**4e**).

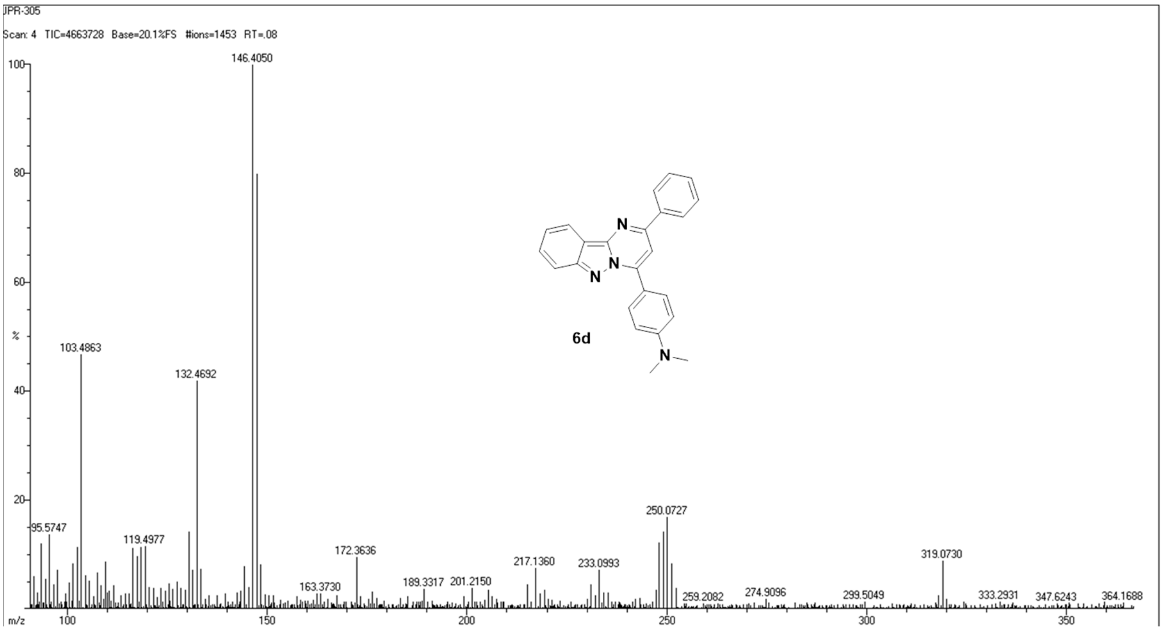

Figure S13. HRMS spectrum of compound (**4e**).

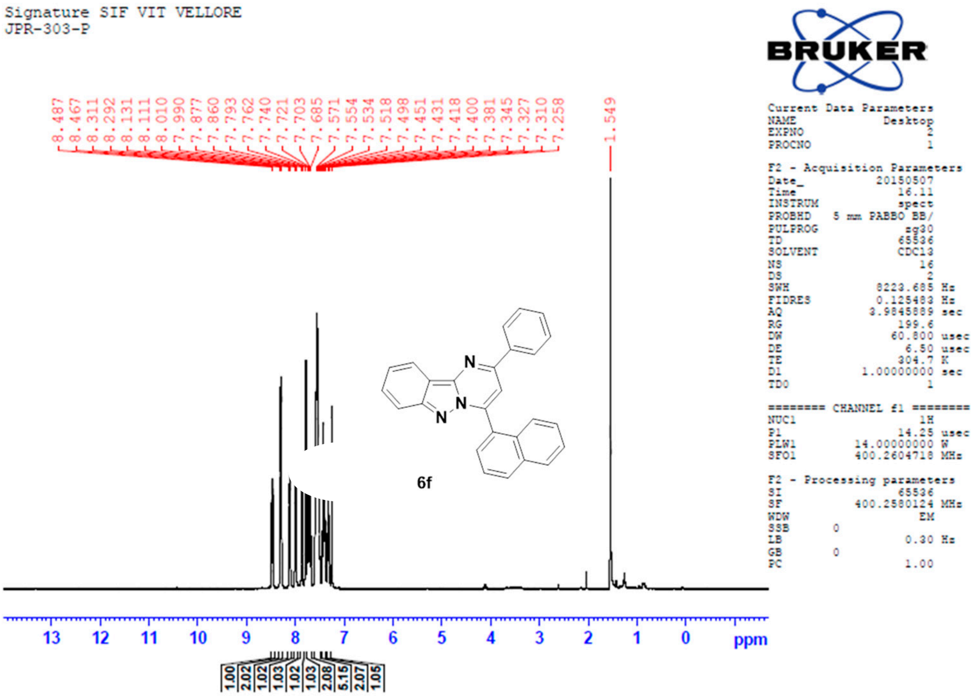

Figure S14. ^{1}H-NMR spectrum of compound (**4j**).

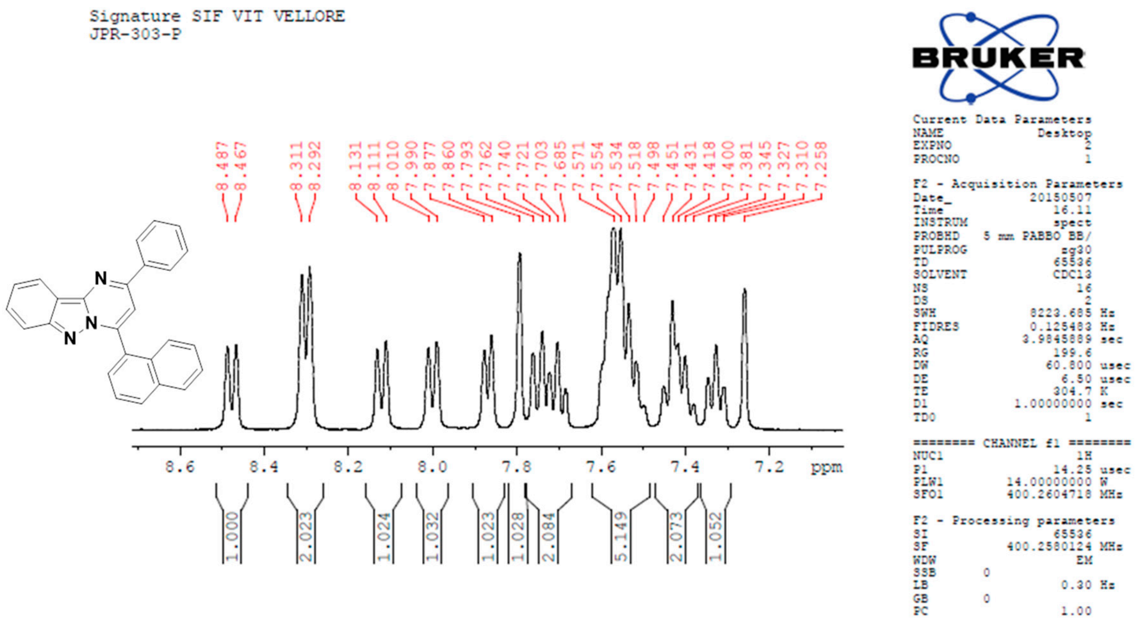

Figure S15. Expanded ^{1}H-NMR spectrum of compound (4j).

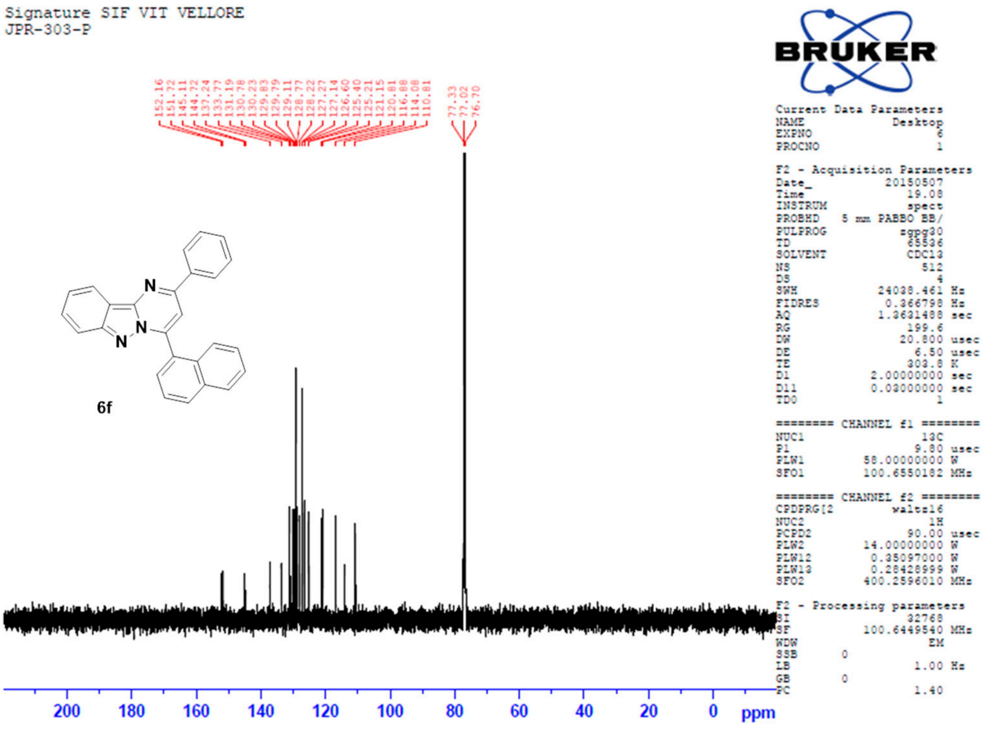

Figure S16. ^{13}C-NMR spectrum of compound (4j).

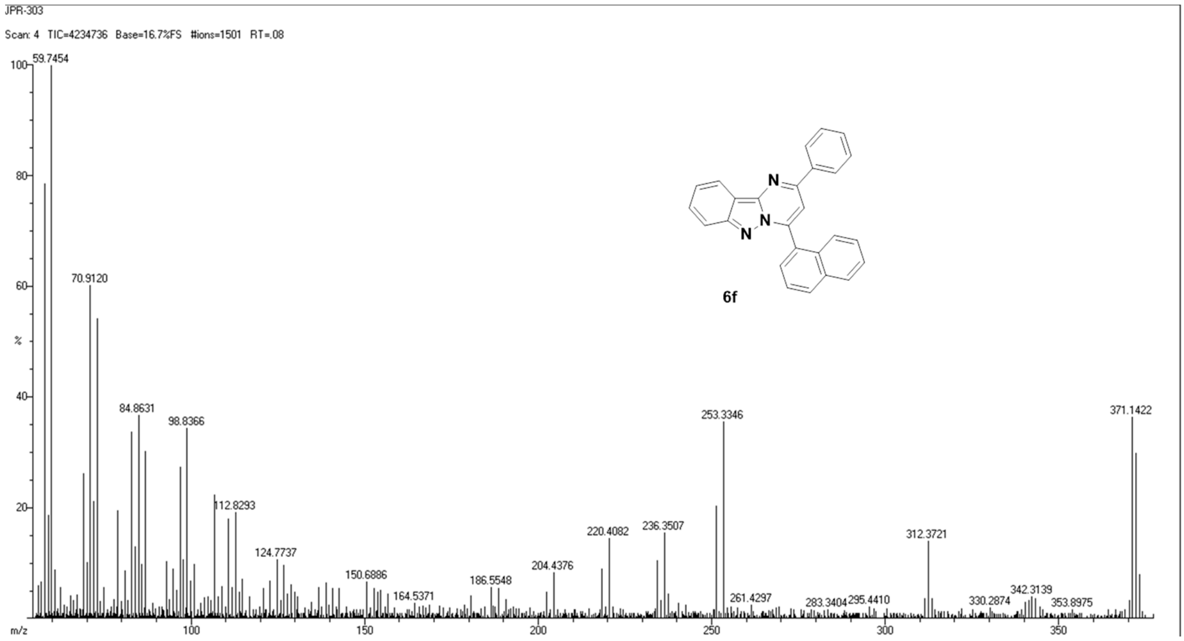

Figure S17. HRMS spectrum of compound (**4j**).

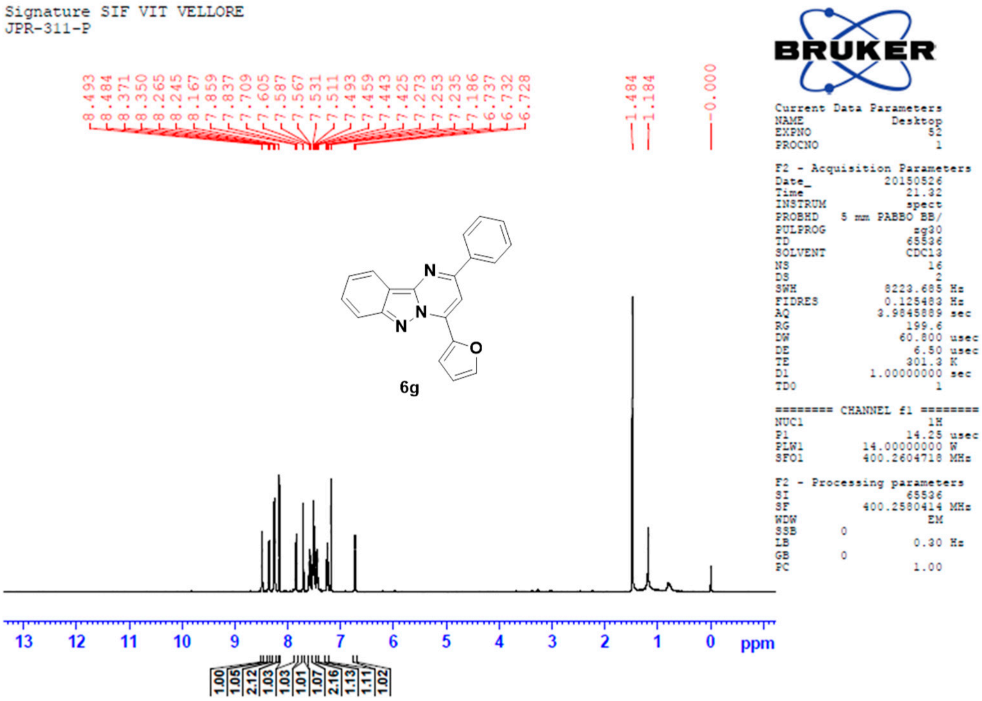

Figure S18. ¹H-NMR spectrum of compound (**4k**).

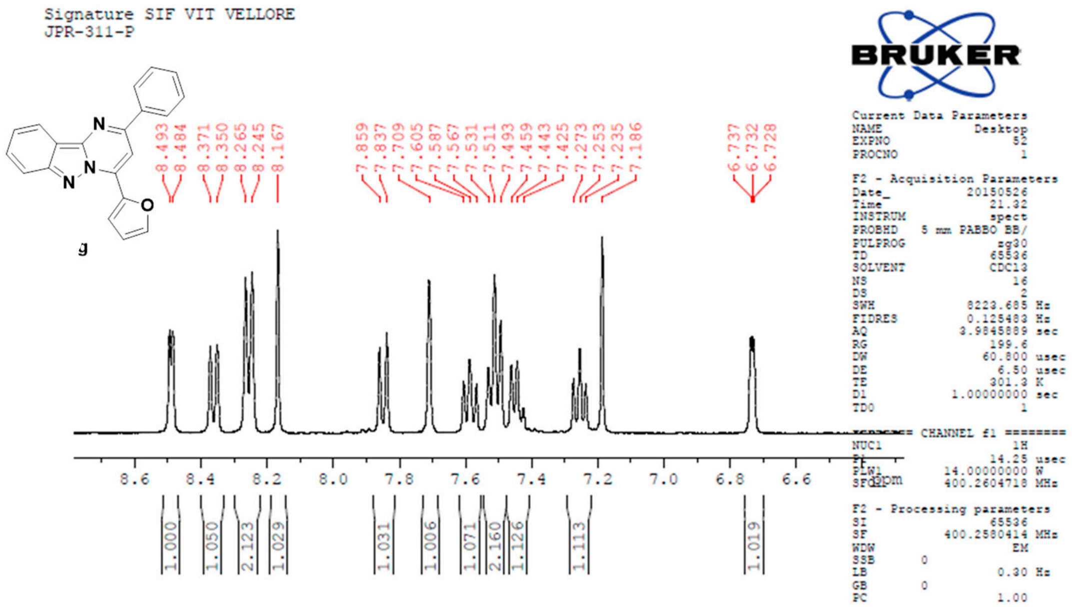

Figure S19. Expanded ^{1}H-NMR spectrum of compound (**4k**).

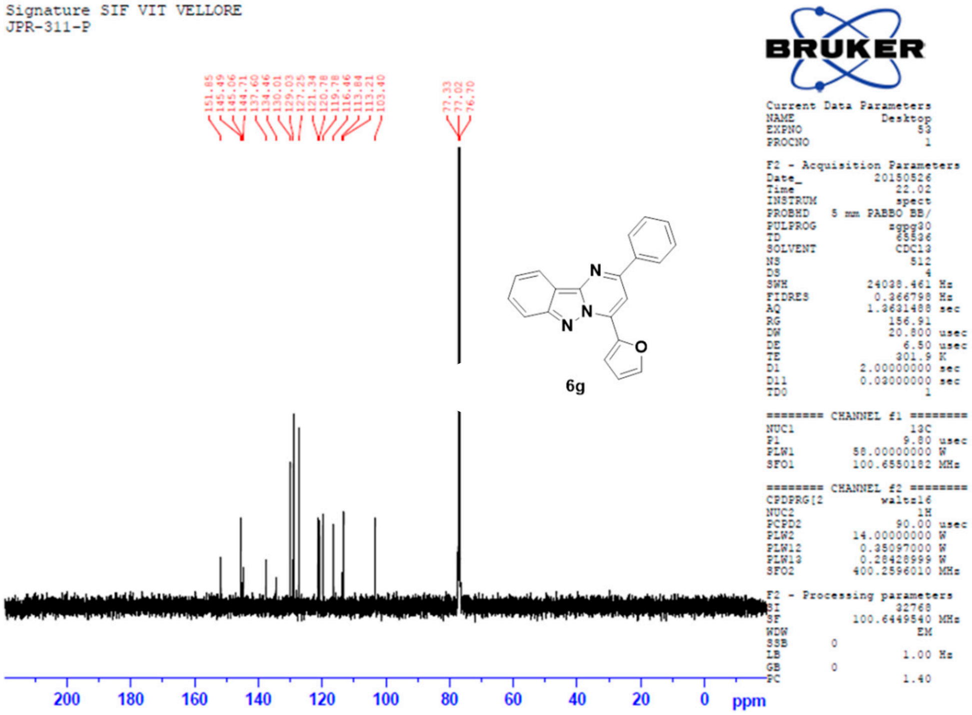

Figure S20. ^{13}C-NMR spectrum of compound (**4k**).

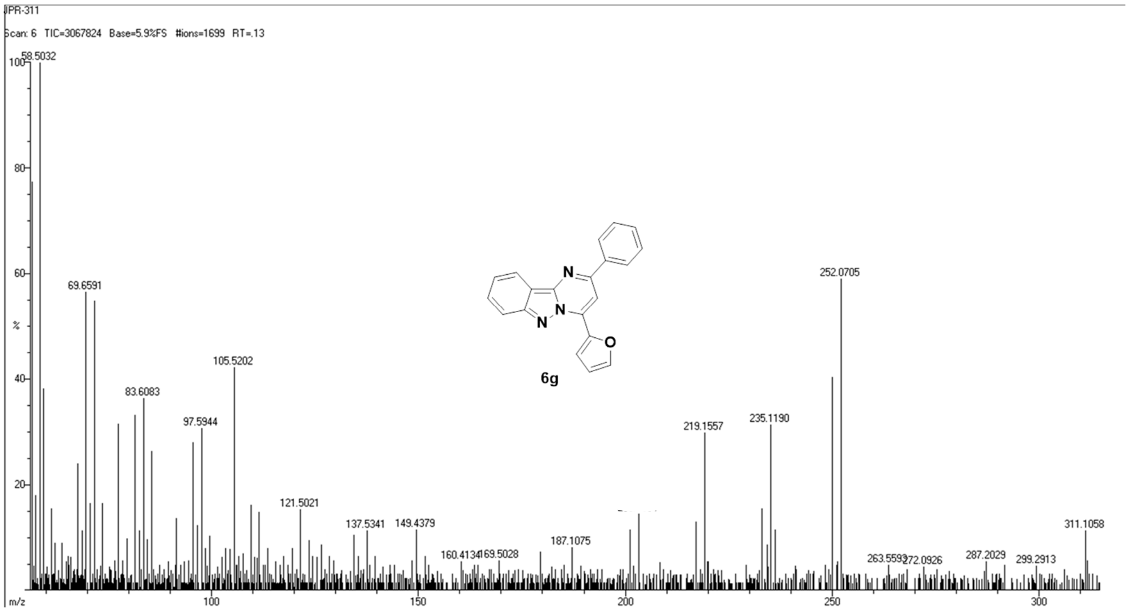

Figure S21. HRMS spectrum of compound (**4k**).

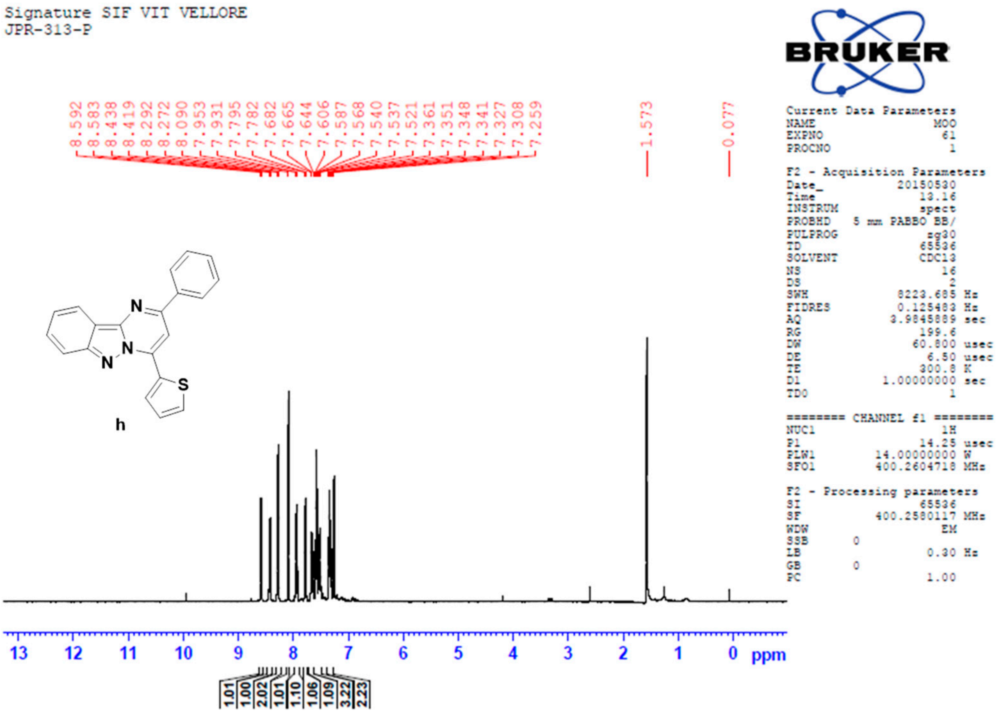

Figure S22. ^{1}H-NMR spectrum of compound (**4l**).

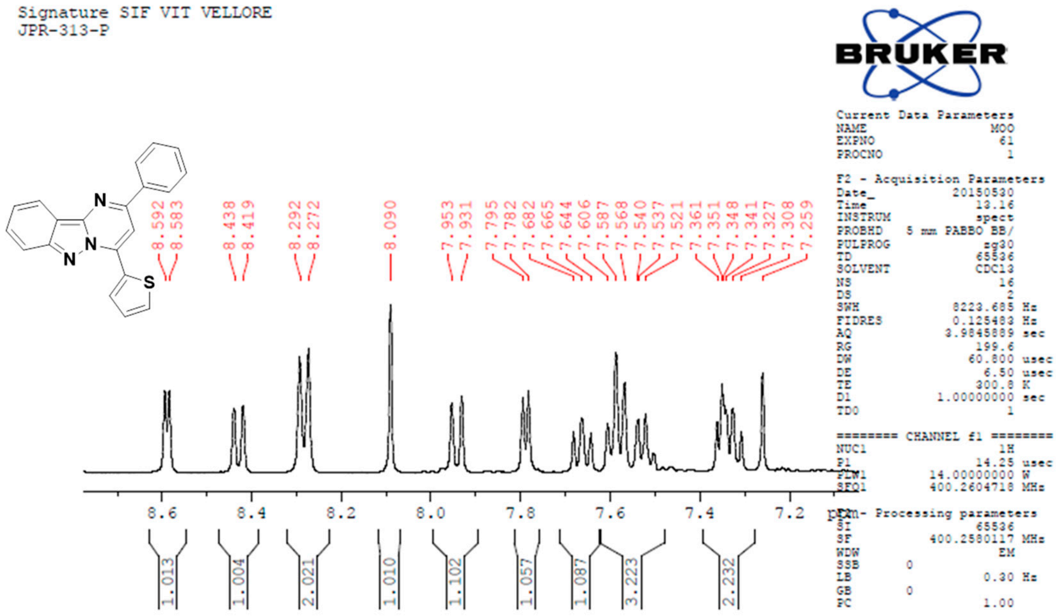

Figure S23. **E**xpanded ^{1}H-NMR spectrum of compound (**4l**).

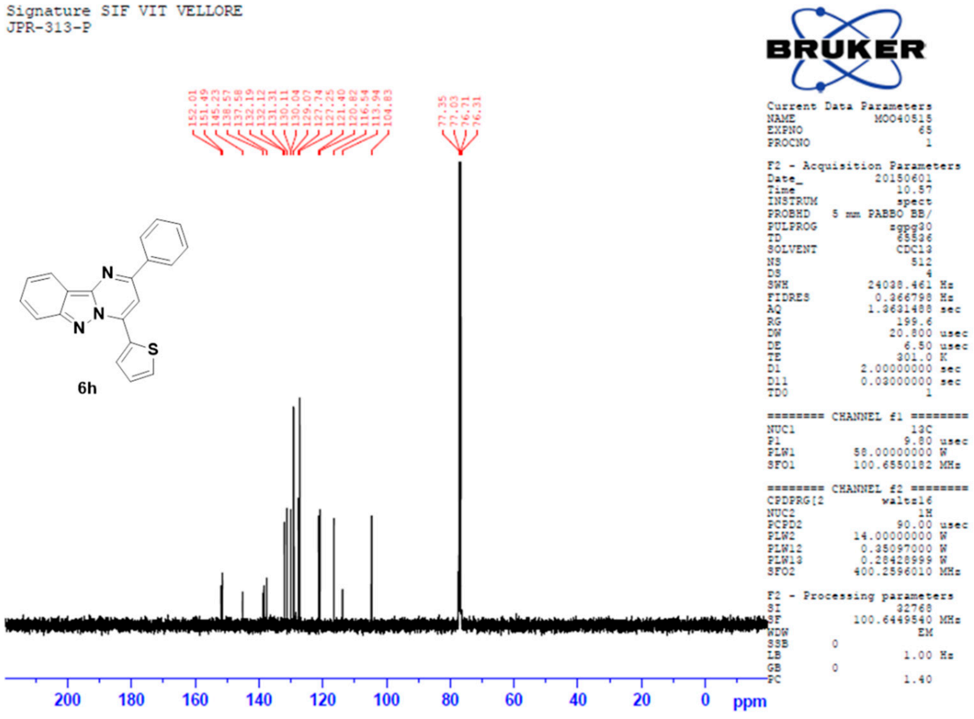

Figure S24. ^{13}C-NMR spectrum of compound (**4l**).

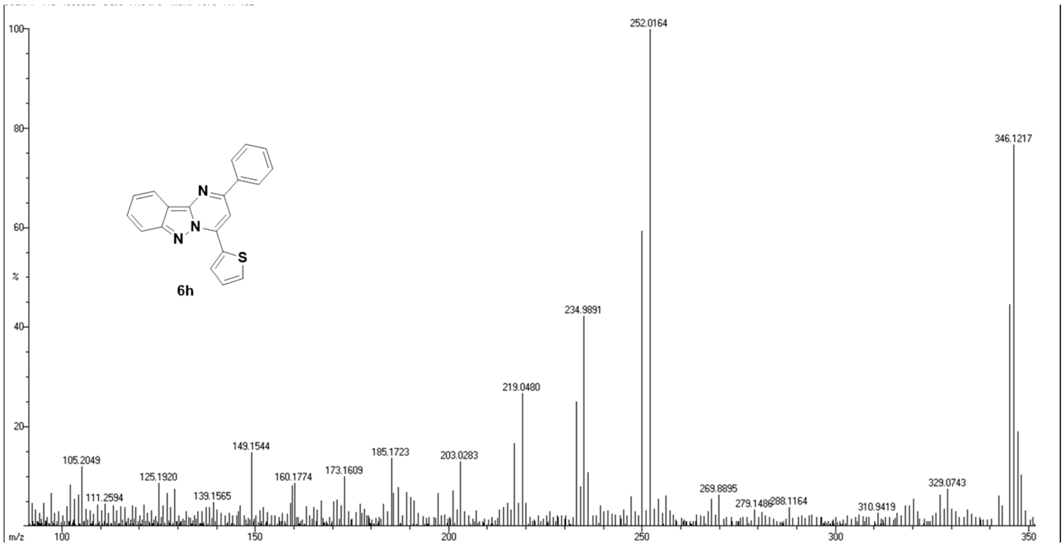

Figure S25. HRMS spectrum of compound (**4l**).

Table S1. IC_{50} values of the free radical scavenging activity of pyrimido fused indazoles.

S. No	Compounds	% of Inhibition		IC_{50}
		0.001 mM	0.002 mM	
1	**4a**	75.56	76.1	46.33
2	**4b**	75.7	76.1	63.25
3	**4c**	75.03	74.09	27.62
4	**4d**	76.03	76.83	31.53
5	**4f**	75.63	63.35	3.08
6	**4g**	80.17	76.36	8.91
7	**4h**	74.76	73.29	17.84
8	**4i**	75.43	73.36	13.28
9	**4j**	77.57	75.28	13.03
10	**4k**	76.9	73.36	8.59
11	**4l**	69.35	68.29	19.25
12	**4m**	74.76	74.56	124.8
13	**4n**	74.69	77.1	9.24
14	**4o**	80.97	74.25	5.60
15	Ascorbic acid	81.8	87.58	4.50
